# Reconstituting protein interaction networks using parameter-dependent domain-domain interactions

**DOI:** 10.1186/1471-2105-14-154

**Published:** 2013-05-07

**Authors:** Vesna Memišević, Anders Wallqvist, Jaques Reifman

**Affiliations:** 1 Department of Defense Biotechnology High Performance Computing Software Applications Institute, Telemedicine and Advanced Technology Research Center, U.S. Army Medical Research and Materiel Command, Fort Detrick, MD 21702, USA

**Keywords:** Merging domain annotations, Domain-domain interactions, Protein-protein interaction networks

## Abstract

**Background:**

We can describe protein-protein interactions (PPIs) as sets of distinct domain-domain interactions (DDIs) that mediate the physical interactions between proteins. Experimental data confirm that DDIs are more consistent than their corresponding PPIs, lending support to the notion that analyses of DDIs may improve our understanding of PPIs and lead to further insights into cellular function, disease, and evolution. However, currently available experimental DDI data cover only a small fraction of all existing PPIs and, in the absence of structural data, determining which particular DDI mediates any given PPI is a challenge.

**Results:**

We present two contributions to the field of domain interaction analysis. First, we introduce a novel computational strategy to merge domain annotation data from multiple databases. We show that when we merged yeast domain annotations from six annotation databases we increased the average number of domains per protein from 1.05 to 2.44, bringing it closer to the estimated average value of 3. Second, we introduce a novel computational method, *parameter-dependent DDI selection* (PADDS), which, given a set of PPIs, extracts a small set of domain pairs that can reconstruct the original set of protein interactions, while attempting to minimize false positives. Based on a set of PPIs from multiple organisms, our method extracted 27% more experimentally detected DDIs than existing computational approaches.

**Conclusions:**

We have provided a method to merge domain annotation data from multiple sources, ensuring large and consistent domain annotation for any given organism. Moreover, we provided a method to extract a small set of DDIs from the underlying set of PPIs and we showed that, in contrast to existing approaches, our method was not biased towards DDIs with low or high occurrence counts. Finally, we used these two methods to highlight the influence of the underlying annotation density on the characteristics of extracted DDIs. Although increased annotations greatly expanded the possible DDIs, the lack of knowledge of the true biological false positive interactions still prevents an unambiguous assignment of domain interactions responsible for all protein network interactions.

Executable files and examples are given at: http://www.bhsai.org/downloads/padds/

## Background

The living cell is a dynamic, interconnected system where proteins interact with each other to facilitate biological processes. Large protein-protein interaction (PPI) datasets have become available due to advances in experimental biology and the development of high-throughput screening techniques. However, while existing data describe thousands of protein interactions, such interactions still constitute only a fraction of all PPIs for a small number of available organisms [1-5]. Moreover, available PPI datasets acquired from different experiments are often seemingly inconsistent with each other, implying that the different methods might produce false positive interactions or fail to identify certain types of interactions [4,6-9]. Here, we attempt to address this seemingly intractable problem by focusing on bioinformatics approaches that use protein domains as fundamental building blocks of protein interactions.

### Domains as protein interaction building blocks

Proteins consist of one or more domains and multiple studies have shown that domain-domain interactions (DDIs) from different experiments are more consistent than their corresponding PPIs, suggesting that domains may be fundamental in mediating physical interactions between proteins [10-12]. Under the assumption that protein interactions are mediated by domain interactions, we can hypothesize that each interaction in a PPI dataset can be converted into a corresponding set of pairwise domain interactions. However, lack of direct experimental evidence for interactions at the domain level means that we can only account for, or explain, a small fraction of known PPIs for any organism using experimentally determined DDIs. Determining the particular domains that physically bind (*i.e.*, mediate) a given PPI based on limited structural information remains a challenge.

**Figure 1:**
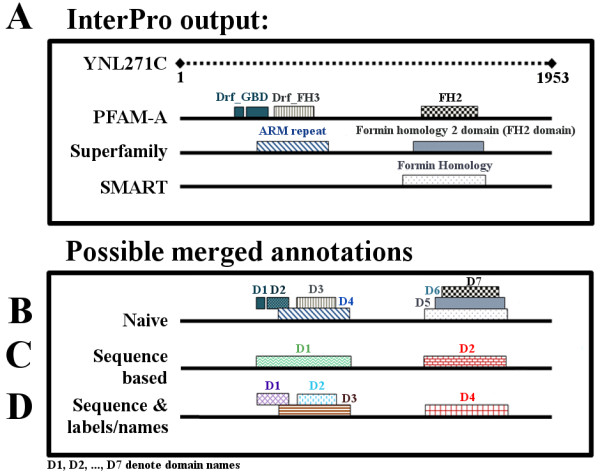
**Evaluation of different protein-domain annotation merging strategies.** (**A**) Using the InterPro database, we obtained seven protein-domain annotations for yeast protein YNL271C from three databases: PFAM [32], Superfamily (SF) [33], and SMART [34,35]. PFAM domains: FH2, Drf*_FH3*, and two *Drf_GBD* domains; SF domains: *Formin homology 2 domain (FH2 domain)* and *ARM repeat*; and SMART domain: *Formin Homology*. (**B**) The naïve domain-merging strategy identified seven unique domains for YNL271C. (**C**) Sequence locations helped identify some of the identical domains (*FH2*, *FH2 domain*, and *Formin Homology*) but was not able to differentiate between different domains that share the same sequence position. (**D**) Taking into consideration both sequence location and domain names/labels, our merging strategy identified four unique domains: *ARM repeat*, *Drf_FH3*, *Drf_GBD*, and a domain consisting of *FH2* domains (*FH2*, *FH2 domain,* and *Formin Homology*).

To address this challenge, we must first characterize the specific protein domains that mediate protein interactions. It is estimated that approximately 80% of eukaryotic proteins and 67% of prokaryotic proteins have multiple domains [13,14]. Most annotation databases characterize each domain family using a small, curated set of amino acid sequences common to representative members. These databases share a significant amount of protein-domain annotation data; however, each database also contains a noteworthy number of unique protein-domain annotations. Some databases, *e.g.*, Conserved Domain Database (CDD) [15] and InterPro [16,17], provide protein-domain annotation information collected from several databases but none provides the capability to methodically merge these annotations (Figure 1A).

Combining data from multiple databases, while addressing annotation inconsistencies, is a non-trivial procedure. For example, a naïve domain annotation data-merging strategy consisting of the aggregation of all annotation data regardless of domain sequence overlaps or domain name/label similarities would increase the average number of hypothetical domains per protein. However, this strategy would also overestimate the total number of domains, because it considers domains that are not identically represented in two different databases as two different domains (Figure 1B). In contrast, considering sequence information as part of the naïve merging strategy, *e.g.*, by aggregating all annotation data that overlap in at least 10 continuous amino acids, would reduce the number of inferred domains per protein. However, such a merging strategy inherently assumes that all domains that overlap in sequence are identical, leading to a small number of merged domains and, likely, an underestimation of the total number of true domains (Figure 1C). The strategy presented here combines sequence locations and name/label information to construct merged domain annotation sets in which the number of domains per protein is not *a priori* over- or underestimated (Figure 1D).

### Domain-based methods for reconstituting whole protein interaction networks

The use of domains as mediators of protein interactions requires the ability to assign domains to all proteins under consideration. However, in the case of multi-domain proteins, it is unclear which particular domains truly mediate a given PPI set, because more than one potential domain pair can account for a single interaction. This uncertainty could lead to predictions of false positive PPIs, as domains identified as mediators of protein interactions account not only for the original PPI set but also for all other protein pairs that contain the same domain pair combinations. Existing computational methods use varying approaches to tackle different aspects of these problems, each with its own set of aims, strengths, and limitations [18-30]. For example, some methods use additional biological information, such as gene expression data, to establish whether a PPI can occur [24,25,29], and others limit the PPI coverage to smaller sets of high-confidence interactions [19,26,27]. An additional promising approach is to use a feature selection algorithm to find a set of DDIs that best discriminate between true and false PPIs [30]. However, these methods are not broadly applicable to non-model organisms or comprehensive enough to include protein interactions on a proteomic scale.

In this regard, reconstitution methods provide a framework that does not *a priori* require additional data and is applicable on a genomic scale to any organism provided a PPI dataset exists [20,21,23]. The aim of these methods is to identify small sets of potential DDIs that reconstitute the complete original set of PPIs. Overall, the aim of the maximum-specificity set cover (MSSC) method [23] is to minimize the number of potential false positive interactions regardless of the number of DDIs used to explain the PPI set, while the aim of the parsimonious approach (PA) [20] and the generalized parsimonious explanation (GPE) method [21] is to minimize the number of selected DDIs regardless of the introduction of false positive interactions. Despite their underlying differences, all three approaches (MSSC, PA, and GPE) have been shown to recover DDIs experimentally identified from structural data. This leads to the observation that true DDIs are not necessarily rare, promiscuous, or parsimonious, but rather are distributed between the extremes. Consequently, a method that reconstitutes protein interactions based on different degrees of rare, promiscuous, and parsimonious DDIs could prove beneficial.

### Our contributions

Here, we investigate how to create merged sets of domain annotations and how to use these annotations to select sets of DDIs that reconstitute large-scale PPI networks using different true positive and false positive selection weights. First, we introduce a novel computational strategy to merge protein-domain annotation data from multiple databases, a needed capability that is not currently available elsewhere. We believe that merging protein-domain annotation data from multiple sources will help ensure a large and consistent domain annotation set for any given organism. Second, we introduce a novel heuristic computational approach, *parameter-dependent DDI selection* (PADDS), which, given a set of PPIs, extracts a small set of DDIs that explains the original set of protein interactions and is not biased towards DDIs with either low or high occurrence counts. The heuristic scoring system for selecting DDIs can be tuned between favoring known interactions (true positives) and penalizing non-observed interactions (false positives). Given that the domain-merging procedure increases the number of domains per protein and, hence, the number of possible domain combinations, PADDS was designed to minimize both the number of false positive PPIs and the size of the extracted DDI set.

## Results and discussion

### Merged domain annotations from multiple databases

Our strategy combines sequence locations and name/label information to construct merged domain annotation sets as detailed in the Methods. Here, we illustrate its application on a well-annotated single-cell organism. We created a merged set of protein-domain annotations for yeast (*Saccharomyces cerevisiae*) using sequences of 5,884 proteins,^a^ downloaded from the Saccharomyces Genome Database (SGD) [31] and yeast annotation data from six commonly used annotation databases: PFAM-A (release 25.0) [32], Superfamily (SF) [33], SMART [34,35], PRODOM [36], TIGRFAM [37], and CDD [15]. To assign protein-domain annotations, we either used curated yeast domain annotations (if available) [32,33] or extracted domain annotations based on an *E-*value threshold of *≤ 10*^*-2*^[15,34-37]. Although approximately 80% of the proteins had at least one domain annotation in one of the databases (Table 1), this level of annotation density cannot be expected for less-studied organisms. Thus, merging protein-domain annotation data from multiple sources will help ensure a maximally large and consistent domain annotation set for any given organism.

**Table 1:** Yeast protein-domain annotation data from six publicly available annotation databases

**Database**	*** N***_***P***_		*** N***_***S***_		*** N***_***O***_	*** N***_***U***_	*** A***_***D***_
	*** n***	** %**	*** n***	** %**			
PFAM-A	4,709	80.0	1,174,333	40.2	2,595	2,553	1.05
SF	3,651	62.1	962,602	33.0	1,355	1,307	0.79
SMART	3,023	51.4	455,523	15.6	392	379	0.66
PRODOM	146	2.5	19,760	0.7	111	111	0.02
TIGRFAM	3,019	51.3	546,226	18.7	2,544	1,944	1.25
CDD	2,210	37.6	560,299	19.2	3,300	731	0.58

A total of 5,884 proteins containing a total of 2,921,809 amino acids were downloaded from the *Saccharomyces Genome Database*[31]. *N*_*P*_, proteins with at least one domain annotation. *N*_*S*_, protein-domain amino acid sequence coverage. *N*_*O*_, number of unique domains in the original database. *N*_*U*_, number of unique domains in the unified database. *A*_*D*_, average number of domains per protein in the unified database.

Table 1 shows that, despite extensive annotation efforts, each database characterized each protein by a small average number of domains. It also shows the variation in the number of domains extracted among the different databases, as well as the variation in the number of proteins with domain annotations.

The content of the final merged domain annotation set does not depend on the order in which we merged the databases. However, to create high-confidence merged annotation sets of different sizes, *e.g.*, merged annotation from two, three, …, six, databases, we first merged the PFAM-A and SF contents because they contain curated domains of high confidence. We selected the merging order of the other four databases randomly and Table 2 shows the database origins of the six merged sets, SET-1 to SET-6.

**Table 2:** Database origin of merged domain annotation sets

**Annotation set**	** Domain annotation databases**
SET-1	PFAM-A [32]
SET-2	PFAM-A, SF [33]
SET-3	PFAM-A, SF, SMART [34,35]
SET-4	PFAM-A, SF, SMART, PRODOM [36]
SET-5	PFAM-A, SF, SMART, PRODOM, TIGRFAM [37]
SET-6	PFAM-A, SF, SMART, PRODOM, TIGRFAM, CDD [15]

Table 3 shows that the merging procedure increased the number of proteins with domain annotation by more than 10%. At the same time, the average number of domains per protein increased from 1.05 to 2.44 (Table 3), approaching the estimated average value of ~3 [10,14]. The final domain annotation set created using the database merging procedure consisted of 4,114 unique domains (Additional file 1). The domain length distribution in this set was similar to the domain length distribution from each of the six original databases (data not shown), and most domains ranged in length between 100 and 300 amino acids.

**Table 3:** Yeast protein-domain annotation data after merging annotations from the six databases

**Domain annotation set**	***N***_***U***_	***N***_***P***_		***N***_***S***_		***A***_***D***_
		***n***	**%**	***n***	**%**	
**Domain-merging procedure**						
SET-1	2,595	4,709	80.0	1,174,333	40.0	1.05
SET-2	2,847	4,964	84.4	1,510,026	51.7	1.33
SET-3	2,806	5,280	89.7	1,653,122	56.6	1.69
SET-4	2,843	5,307	90.2	1,663,269	56.9	1.69
SET-5	4,182	5,392	91.6	1,735,533	59.4	2.55
SET-6	4,114	5,395	91.7	1,756,481	60.1	2.44
**Naïve domain merging**						
SET-6-NB	10,297	5,395	91.7	1,756,481	60.1	5.77
**Domain merging based solely on sequence overlap**						
SET-6-SB	1,492	5,395	91.7	1,756,481	60.1	1.32

Database sets SET-1 through SET-6 are defined in Table 2. SET-6-NB (naïve merging) contained the union of unique domain annotations from the six databases used in SET-6. SET-6-SB contained merged domain annotations from the same six databases as in SET-6, but domains in this set were merged only if their sequences overlapped and they shared at least ten common amino acids (*i.e.*, domain labels were not considered). *N*_*U*_, number of unique domains. *N*_*P*_, proteins with at least one domain annotation. *N*_*S*_, protein-domain amino acid sequence coverage. *A*_*D*_, average number of domains per protein.

### Evaluation of the protein-domain annotation merging strategy

To evaluate the merged domains, we compared our results to those obtained with two simple alternative strategies: a naïve domain-merging strategy (SET-6-NB) and a naïve domain-merging strategy that takes into account sequence overlaps (SET-6-SB). Because the number of original domains is constant, all three merged sets (SET-6, SET-6-NB, and SET-6-SB) yielded the same number of proteins with domain annotation. However, their final domain annotations resulted in different numbers of unique domains, as well as different average numbers of domains per protein (Table 3). SET-6-NB consisted of over 10,000 unique domains, with an average number of 5.77 domains per protein. This set considerably overestimated the total number of unique domains, as many of its 10,000 domains represented the same domain with a slightly different label. For example, the naïve merging strategy would consider the *formin homology 2* domain represented in three annotation databases (PFAM-A, SF, and SMART) as different domains, because their domain labels and sequence locations are not identical (see Figure 1B). By merging annotations that overlap in at least 10 continuous amino acids, SET-6-SB reduced the number of unique domains to 1,492, as well as the average number of domains per protein to 1.32. Although the average number of domains per protein was greater than the average number for any of the original databases, the total number of unique domains was underestimated. For example, the sequence location of *ARM repeat* overlaps with the sequence location of *Drf_FH3* and *Drf_GBD* domains (see Figure 1C) and this strategy would merge the *ARM repeat* with *Drf_FH3* and also with *Drf_GBD*. This would result in a merged domain that consists of the three original domains, *ARM repeat*, *Drf_FH3*, and *Drf_GBD*, even though these three domains are different and should not have been merged. Our merging strategy does not suffer from these issues, as it distinguishes between the same and different domains that cover the same sequence location based on their domain labels (see Figure 1D).

These results showed that our protein-domain-merging strategy did not overestimate or underestimate the number of domains per protein. However, this does not necessarily imply that the merged domain annotation is biologically more relevant. To this end, we compared our merged protein-domain annotations to the recently released high-confidence annotations from the PFAM-A database (PFAM release 26.0). To assess the amount of correctly retrieved annotations from our merged set, we compared them to the following two independent subsets of the new PFAM release: *1*) a set of new domain annotations that replaced annotations from the previous PFAM release (PFAM release 25.0) and *2*) a set of new domain annotations that did not exist in the previous PFAM release but have a corresponding annotation in the merged dataset.^b^ The comparison procedure consisted of two steps. First, for each new domain annotation, we found one or more merged domain annotations that covered the same protein sequence location. Then, we manually compared domain labels and descriptions between the new domain and the merged domains. Out of 17 new domain annotations in the first subset and 274 new domain annotations in the second subset, we found 13 (76%) and 202 (71%) annotations, respectively, in our merged dataset (Additional file 2). Because these account for >70% of the new PFAM-A annotations, it demonstrates the benefits of the proposed domain-merging strategy.

### Use of annotation-based domains to reconstitute protein interaction networks

The introduction of a more complete set of domain annotations across all interacting proteins in a genome would allow for the enumeration of all domain interactions that could account for an original set of PPIs. Furthermore, this would also allow for a comprehensive evaluation of DDIs and identification of an optimum DDI set. However, this process has the disadvantage of exponentially increasing the number of domain combinations. To circumvent this problem, our PADDS method enumerates only a subset of DDI combinations and evaluates each one of them based on the following two criteria: *1*) the number of DDIs used to account for the observed PPIs and *2*) the number of non-observed PPIs (*i.e.*, false positives) introduced by the combination of DDIs. As detailed in the Methods, this selection depends on the value of the parameter that specifies the true/false positive biases, denoted as *α*; *α* ε [*0.0, 1.0*], where an *α* of 0.0 favors observed protein interactions and an *α* of 1.0 maximally penalizes non-observed interactions. We first used PADDS to investigate the choice of selecting different values of the parameter *α* on retrieved DDIs. Here, the DDIs were constructed from a study containing multiple organisms, but with protein-domain annotations from a single database. The PADDS-extracted DDIs were compared to other methods and validated using the iPFAM [38] and DOMINE [39,40] databases of known and predicted DDIs. We then applied the algorithm to extract DDIs from a high-confidence yeast PPI dataset using merged domain annotations. We compared the results from our analysis to those of existing reconstitution methods on the same datasets.

### Multiple organism PPIs characterized by a single domain annotation database

To determine the consequences of favoring true positives or penalizing false positives, we examined the ability of PADDS to generate different sets of DDIs that can reconstitute a diverse set of PPIs from multiple organisms for different values of *α*. We applied PADDS to a collection of PPIs from 68 different organisms as assembled by Riley *et al*. [27]. In order to compare our results on this dataset to the GPE method, previously identified as giving the best reconstitution results on this dataset [21], we converted all domains to the same PFAM-A supra-domain annotations used by GPE [21]. We identified 10,025 proteins with PFAM-A supra-domain annotations and 20,625 PPIs where both interacting proteins had at least one domain annotation. This dataset yielded a total number of 26,113 potential DDIs that could be used to reconstitute all PPIs and the average number of domains per protein for this dataset was 1.37.

**Figure 2:**
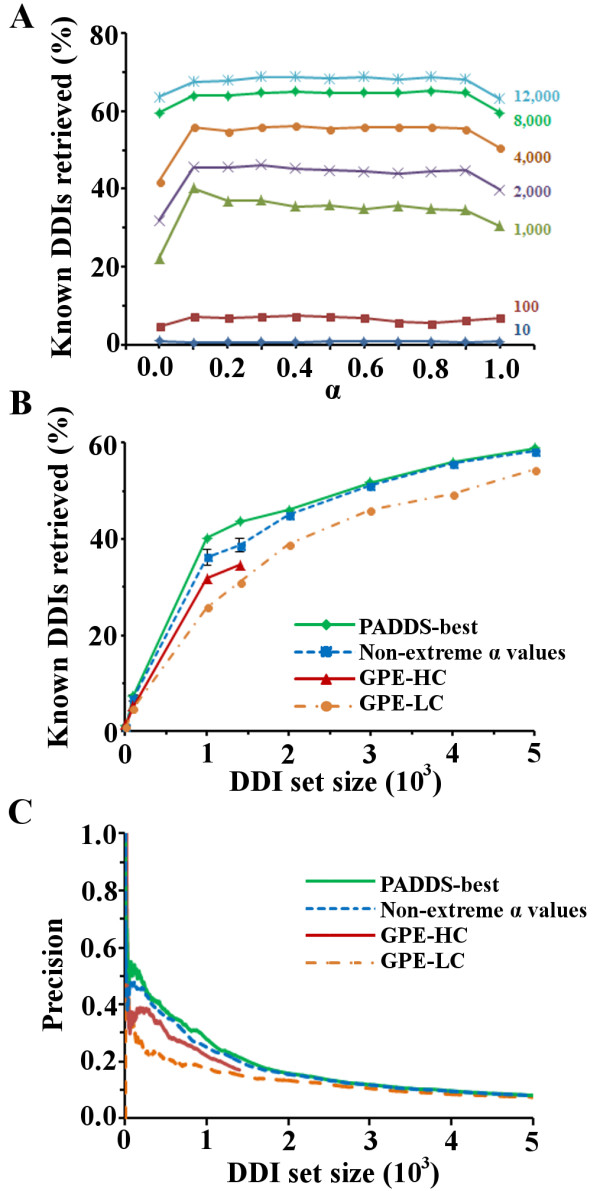
**Enrichment of *“known” *(iPFAM) domain-domain interactions.** Evaluation of the top-scoring domain-domain interactions (DDIs) extracted by the *parameter-dependent DDI selection* (PADDS) and the *generalized parsimonious explanation* (GPE). (**A**) The fraction of known DDIs in the iPFAM database [38] retrieved by PADDS as a function of *α* and the number of top-scoring DDIs. (**B**) Comparison of the percentage of retrieved iPFAM DDIs using PADDS and GPE as a function of top-ranked DDI sets (*i.e.*, recall). (**C**) Comparison of the fraction of retrieved iPFAM DDIs using PADDS and GPE as a function of the iPFAM DDI set and top-ranked DDI sets (*i.e.*, precision). For the GPE sets, we used the DDI rank information provided with the published data that includes their designated high-confidence (GPE-HC) and low-confidence (GPE-LC) sets [21]. We have also indicated the best results achievable with any *α* value, typically achieved for *α = 0.1*.

For each *α* used in PADDS to extract the DDI sets (Additional file 3), we ranked the DDIs based on their corresponding benefit values (see Methods). We evaluated each set of top-scoring DDIs for enrichment of DDIs detected in crystal structures available in the iPFAM database (denoted as “known DDIs”) [38]. Out of 26,113 potential DDIs from the Riley dataset, 691 DDIs were present in the set of known DDIs [20]. Figure 2A shows the fraction of known DDIs retrieved for different values of *α* in different top-ranked DDI sets. The overall number of extracted known DDIs did not increase linearly with the number of DDIs analyzed, and the total retrievable number was less than 70% of the known set. Additionally, the number of known DDIs retrieved varied in a non-linear fashion with *α*, indicating that the extraction procedure was sensitive to the selection weights for both observed and non-observed interactions. These observations imply a non-trivial solution to the optimal DDI extraction problem. We also noted that the largest number of known DDIs were always retrieved in sets for which *α* was not at its extreme values of *0.0* or *1.0*. For the small to intermediate size sets between 1,000 to 4,000 analyzed DDIs, the maximum retrievable number occurred at *α* values ~*0.10*.

Figure 2B, Figure 2C, and Additional file 4: Table S1 show the difference in retrieving known DDIs between PADDS and the published results using GPE methods. For PADDS, we show both the best results using selected *α* values and average results using non-extreme values of *α*. For this dataset, PADDS was more successful (13% – 27%) than the best GPE method in the majority of the *α* selections away from the extreme values. This implies that the ability to modulate the preference for known interactions and tolerance of non-observed interactions was an important factor in the process of DDI extraction and the overall ability to extract known DDIs. While there is always a dataset dependency on these results, it was also clear that relaxing either extreme selection (*α = 0.0* or *α = 1.0*) retrieved more known DDIs (Figure 2A).

Although DDI extraction can be optimized for each dataset by varying *α*, one cannot in all cases independently determine an optimal *α* value. Hence, we were also interested in the robustness of the algorithm and, in particular, evaluating extracted DDIs that are independent of *α*. We used the DOMINE database as a comprehensive source of known and predicted DDIs derived from multiple sources [39,40] to construct DDIs (Additional file 4: Validation of extracted core DDIs section and Additional file 4: Figure S1). The analysis showed that there was a large overlap among the sets of extracted DDIs for different values of *α*, indicating robustness of the algorithm to choices of *α*. Furthermore, the PADDS algorithm was capable of providing parameter-independent and unique DDI predictions not derivable from high-confidence results of other computational procedures. To further characterize PADDS-extracted DDIs, we next examined the high-confidence protein interaction network from a single organism (yeast) with our merged domain annotations.

### Single organism PPIs characterized by multiple annotation databases

To evaluate the influence of the underlying set of PPIs and protein-domain annotation data on the DDI extraction process, we reconstructed a set of high-confidence yeast PPI data created by the Interaction Detection Based On Shuffling (IDBOS) procedure at a 5% false discovery rate [8,41]. We have previously shown that this dataset identified binary interactions as well as, or better than, the high-confidence consolidated yeast two-hybrid set or other high-confidence datasets based on affinity purification followed by mass spectrometry [8,41]. The IDBOS dataset consists of 8,401 PPIs between 1,295 proteins. For protein-domain annotation of the IDBOS dataset, we used our merged protein-domain annotation data (SET-1 to SET-6) as described above. The average number of domains per protein for the IDBOS dataset was 2.69. Additional file 4: Table S2 shows the complete statistics for the domain annotations in the IDBOS dataset.

### Evaluating domain interactions for high-confidence yeast protein interactions

We evaluated the merged domain annotation sets using three reconstitution methods: PADDS, MSSC, and GPE. We used PADDS with parameter *α* ε [*0.0, 1.0*] in *0.1* increments, ranked the extracted DDIs based on the corresponding benefit value, and extracted the corresponding ranked data for MSSC and GPE (see Methods). Although, by construction, all obtained DDI sets accounted for all original PPIs, different methods yielded DDI sets of different sizes for each of the six domain annotation schemes, with PADDS consistently extracting the smallest sets of DDIs. Additional file 4: Figure S2, Additional file 4: Table S3, and Additional file 4: Table S4 provide the complete results of this analysis. However, despite of their aim to minimize the number of false positives, all three methods identified a much larger number of novel (predicted) PPIs than what could be expected to occur in a living cell [1,2,4,5]. Even if we assume that all predicted interactions represent plausible physical interactions between proteins, *e.g.*, a specified PPI would occur if two proteins were in close proximity, it is likely that in their native environment they are under additional biological regulation. Thus, one cannot assume that all proteins that contain interacting domains will necessarily interact within the cell, due to the existence of alternative regulatory mechanisms that control these interactions [42].

To evaluate the performance of the different reconstitution methods on different domain annotation sets, we investigated the ability of each method to extract DDIs that accounted for the given PPIs while limiting the number of false positive PPIs. For this calculation, we defined the set of true non-interacting protein pairs as the set of all pairwise protein interactions minus the known true interaction set [18,20,30], see Methods. Based on these definitions, we could then ascertain true and false PPI predictions for each extracted set of DDIs and construct the corresponding Receiver Operating Characteristic (ROC) curves from an analysis of true positive and false positive rates. PADDS outperformed the other two methods for all six annotation sets (Additional file 4: Figure S3 and Additional file 4: Figure S4). The largest differences were most evident for the larger annotation sets, *e.g.*, SET-6, where the other methods lack PADDS’s flexibility to extract a small number of DDIs while limiting the introduction of non-observed interaction.

### PADDS increases diversity of DDIs when provided with sufficient amounts of annotations

**Figure 3:**
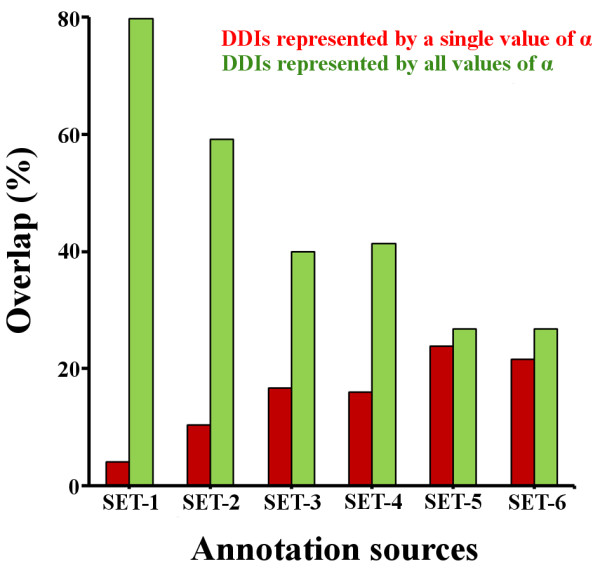
**Overlap between extracted domain-domain interaction sets for different values of parameter *α*.** The graph indicate fractional overlaps between sets of extracted domain-domain interactions (DDIs) for the six different domain annotation schemes defined in Table 2, for different sets of *α* values. As the underlying set of PPIs, we used a high-confidence yeast PPI data set created by the Interaction Detection Based On Shuffling (IDBOS) procedure at a 5% false discovery rate [8,41].

To investigate the relationship between the size of the domain annotation sets and the obtained results, we compared the set of DDIs (accounting for the IDBOS set of PPIs) extracted by PADDS for different values of *α*. We found that, for SET-1, approximately 80% of the DDIs were represented in all extracted sets and were not dependent on the particular value of *α* (a similar result was observed in the multi-organism study). Figure 3 shows that, with an increasing amount of domain annotation data, the number of DDIs represented in all extracted sets decreased, and for SET-6 only ~30% of the DDIs were represented in all sets. In contrast, we observed an increased percentage of DDIs represented by a single value of *α* with larger annotation sets, implying that this parameter introduced significant variations among the extracted DDI sets when more domain annotation data were available. These observations suggest that, for limited amounts of domain annotation data, computational methods are forced to select particular DDIs, as these DDIs are the only ones that could account for certain PPIs. Using additional domain annotation data removed this bias, as more than one DDI accounted for a larger number of PPIs.

In summary, PADDS extracted the smallest set of DDIs for this extensively annotated high-confidence network. However, similar to other methods, regardless of how we biased our benefit score in the extraction process or how efficient PADDS was in extracting true positives, a large number of non-observed PPIs resulted from these DDI selections.

## Conclusions

Proteins consist of one or more domains, and physical interactions between proteins arise from interactions between their specific domains. Given that there is more consistency in DDIs detected from different experiments than in the corresponding PPIs, the hope is that an in-depth analysis of DDIs would improve our understanding of PPIs and give us better insights into cellular function, disease, and evolution. However, determining which particular DDI mediates any given PPI is challenging, because currently available experimental DDI data accounts for only a small fraction of all existing PPIs. In this paper, we present two contributions to the field of domain interaction analysis.

First, we introduced a novel computational strategy that systematically merged domain annotation data from multiple databases; a needed capability that is not currently available elsewhere. By combining sequence locations with domain name and labeling information, our merging strategy was less likely to grossly overestimate or underestimate the number of domains per protein. We showed that merging domain annotations from six different databases increased the average number of domains per proteins, bringing it closer to the estimated true value. We believe that our merging strategy can ensure a large and consistent domain annotation set for any given organism.

The second contribution detailed here is the development of PADDS, a novel computational method that, given a set of PPIs, can identify a small set of potential DDIs that account for the provided set of PPIs and is not biased towards DDIs with low or high occurrence counts. We showed that PADDS was more successful in extracting known DDIs, *i.e.*, DDIs that have been determined experimentally from crystal structures, than the MSSC method and the current best reconstitution method, GPE.

It was also noteworthy that the choice of *α* value influences the number of known DDIs retrieved. For the PPI dataset aggregated from multiple organisms from different sources and annotated by PFAM only, we retrieved the largest number of known DDIs for small *α* values in the range of *0.05-0.10*. We interpreted this to indicate that a small tolerance of false positives in the PPI reconstitution procedure relaxed constraints in the DDI selection process sufficiently enough to garner additional known DDIs, yet avoiding overwhelming the solution with too many non-observed interactions. This result also hints that the hypothesis that all protein interactions must strictly be composed of pairwise domain interactions could be relaxed. We further found that increased amounts of domain annotation data increased the diversity of DDIs that could account for a single PPI. As a result, for the densely annotated high-confidence yeast PPI network, we found that less than 30% of the extracted DDIs were present in all extracted sets. This last observation indicates that, once we have a sufficient amount of annotation data, more diverse DDI sets can be used to reconstitute PPI sets equally well. As currently available reconstitution methods identify only a single set of DDIs that account for a given set of PPIs, a method that is able to identify multiple DDI sets without *a priori* bias towards DDIs with either low or high occurrence counts is a needed capability that is not currently available elsewhere.

## Methods

### Domain annotation and interaction datasets

We used a number of available genome-scale annotation databases that contain domain information. Each database collates information based on different objectives and criteria. PFAM-A contains manually curated protein families and provides assignments of high-confidence domain annotations through family-specific domain gathering thresholds [32]; we used PFAM-A release 25.0. SF contains structural and functional domain annotation, derived from the structural protein domains from the SCOP (Structural Classification of Protein) database [33]. SMART provides annotation of signaling domains [34,35], while PRODOM [36] and TIGRFAM [37] provide protein domain family annotations constructed automatically by sequence homology. CDD, which provides functional protein annotations, also lists domain annotations using multiple sequence alignment models for domains and proteins, as well as curated, structural domains and domains imported from a number of other protein-domain annotation databases (e.g., PFAM, SMART, and TIGRFAM) [15].

Similarly, we used three databases to verify the extracted domain interactions: *1*) the iPFAM database that contains domain-domain interactions obtained from the PDB structures [38], *2*) the domain-domain and peptide-mediated interactions of known 3D structure database (3DID) [43,44], and *3*) a comprehensive collection of known and predicted DDIs (DOMINE) [39,40].

### Protein – domain annotation merging strategy

**Figure 4:**
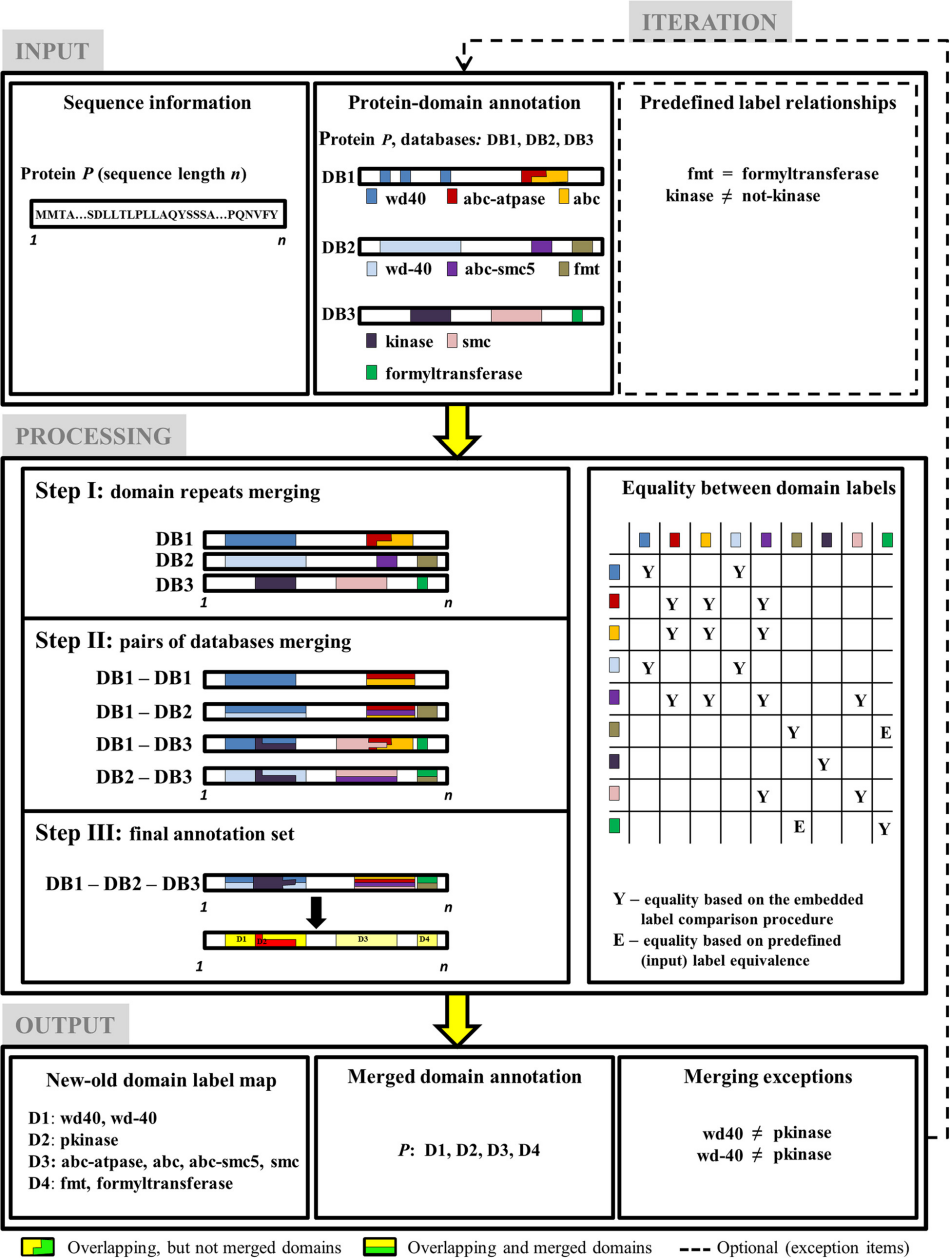
**Protein-domain annotation merging procedure.** An illustration of the computational procedure used to merge protein-domain annotation data from multiple databases for a single protein *P* (consisting of *n* amino acids) and domain annotation data from three databases: DB1, DB2, and DB3. INPUT: Protein sequences and protein-domain annotations from one or more databases. PROCESSING: The annotation data were merged in three consecutive steps. In Step I, tandem domains within each protein (and for each database) were merged and represented as a continuous domain with the same domain label as the tandem domains. In Step II, annotation data between all pairs of databases were merged. In Step III, all pairs from Step II were merged into a final annotation set. In this step, new domain labels were assigned to the sets of merged domains. OUTPUT: The output of the annotation merging procedure consists of *1*) a set of new (merged) domain labels assigned to the protein, *2*) a mapping between the new and original domain labels, and *3*) a list of merging exceptions. Based on these lists, one may (re)define sets of labels that should be treated as equivalent or non-equivalent and iterate through the complete domain annotation merging procedure (ITERATION).

Our merging strategy combines protein-domain annotation data through the following three consecutive steps (Figure 4):

Step I – Domain repeats merging procedure

In the first step, we merged domain repeats within each database. Domain repeats represent two or more domains from the same domain family that appear in tandem [45]. Different proteins may have domain repeats that consist of a different number of domains from the same domain family. In annotation databases, domain repeats are represented either as a set of domains that appear in tandem or as a single domain that corresponds to the union of the tandem domains. Our procedure aimed to represent each domain repeat as a single domain. This ensured a uniform representation of all domain repeats and ultimately removed inconsistencies among databases. To this end, for each database and for each protein of interest, our method flagged domains with identical labels and assigned them to a single domain. The new domain inherited all labels of its members. Furthermore, its sequence was represented by a continuous amino acid sequence containing both the member domains and the amino acid sequences between the domains (Figure 4, Step I).

Although tandem domains that consist of different numbers of domains from the same family may have different functional roles, our current implementation did not distinguish between them. We chose this strategy because the domain annotation data retrieved from most annotation databases depend on an *E*-value threshold and, hence, it was not possible to accurately and indisputably determine how many domains appear in tandem. Furthermore, the *E*-value threshold could also influence the length of the amino acid sequence between tandem domains that were merged together. For this reason, we did not impose a limit on the minimum or maximum sequence length between tandem domains in the merging procedure.

Step II – Merging annotation data between pairs of databases

In the second step, we merged annotation data between each pair of databases, including each database with itself. This step ensured that all possible domain pairs were considered and it removed any possible effect of the order in which domains and databases were merged. In this step, for each protein, we grouped domain annotations into sets such that domains within a set had equivalent domain labels and overlapped with approximately the same segment of the protein sequence, *i.e.*, it matched at least ten continuous amino acids. The final merged domains were not sensitive to the examined threshold variations ranging from 1-30 amino acids. Domain annotations within each set were merged into a single domain. The new domain inherited all domain labels of its members, *i.e.*, it became a multi-label domain. Furthermore, the sequence of the newly defined domain represented a continuous amino acid sequence that consisted of the union of all amino acid sequences of the member domains (Figure 4, Step II). By using a combination of domain labels and domain sequences in the merging procedure, the method ensured that potentially different domains that covered approximately the same segment of a protein sequence were not merged together.

To determine if two domain labels were equivalent, we first represented each one of them as an array of words contained within each label. Because domain labels often contain general common/trivial words (*e.g.*, *a*, *the*, *domain*, *family*, *like*, *member*, *of*, *via*, *within*), these words were excluded from the domain labels. Next, we compared all words from the first array to all words from the second array to determine whether they consisted of identical words or words that were contained within each other (*e.g.*, “*kinases*” and “*pkinase*”). If such a pair of non-trivial words was detected, the two corresponding domain labels were considered equivalent. Clearly, this method of determining the equivalence of two labels does not guarantee a correct outcome. Therefore, our method allows users to specify pairs of labels that should be considered equivalent as well as pairs of labels that should be considered non-equivalent (Figure 4, “Predefined label relationships”). This functionality also overcame problems that arose from labeling-scheme variations that were not always recognized by computational procedures for string comparison. For example, the labels “*fmt*” and “*formyltransferase*” are equivalent, where the first word represents an abbreviation of the second. However, these words are neither the same nor contained within each other, and their equivalence cannot be detected solely by string comparison. A computational procedure that could detect such equivalence based on string manipulation would yield many false positives and was not pursued.

Step III – Creating a final annotation set

In the third step, all pairs of domains from all databases in the second step were merged into a final annotation set. The merging procedure was similar to the one from Step II. Here, however, each domain annotation set already contained some merged domains from Step II. Therefore, for merged domains, the representative sequence was the sequence derived in Step II and the representative label was a multi-label annotation, whereas, for domains that had not been merged, the original label was used as their annotation. For each protein, we grouped the domain annotations into sets such that domains within the same set had equivalent domain labels and overlapped with the same sequence locations. Then, we merged the domain annotations within each set into a single domain. Finally, we assigned new domain labels to each set of merged domains (Figure 4, Step III).

By merging all joined pairs from Step II, it was possible to detect additional overlap between domain labels that were not detected in Step II. For example, given three domains with labels “*abc-smc5*,” “*abc-atpase*,” and “*smc*” from three different databases, the computational procedure in Step II would identify the domain labels “*abc-smc5*” and “*abc-atpase*” as equivalent, the labels “*abc-smc5*” and “*smc*” as equivalent, and the labels *“abc-atpase”* and *“smc”* as not equivalent. Only the first and second pairs of domains would therefore be merged, assuming that all three domains covered approximately the same sequence stretch. However, in Step III, the computational procedure would determine that the merged domain “*abc-atpase abc-smc5*” was equivalent to the merged domain “*abc-smc5 smc*,” and that the “*smc*” and “*abc-atpase*” domains would thus be identified as equivalent, even though this was missed in Step II (Figure 4).

For all string (word) comparison procedures, we used string comparison algorithms available in a standard *C+*+ library.

For each protein of interest, our method outputs the newly assigned domain labels and their corresponding sequence locations. Additionally, the procedure provides a list (dictionary) that contains mappings between the new domain labels and labels from the original databases, as well as a list of domain labels that overlapped in sequence but were not similar enough to be merged. These lists can be used to redefine a set of labels that should be treated as the equivalent or different (Figure 4).

### Definition of true and false positive/negative predicted PPIs

In this work, we have adapted an operational definition of true and false PPI predictions based on what is known about a given protein interactions network. Given a set of *n* proteins and *m* known, experimentally detected pairwise interactions among these proteins (the interacting set), we defined the set of non-interacting protein pairs as the set that includes all pairwise PPIs among the *n* proteins, except for the known interactions. Hence, the number of non-interacting PPIs is given by $\;\left(\begin{array}{c} n\\ 2 \end{array}\right)-m$[18,20,30]. We then defined a true positive (TP) PPI prediction as a predicted PPI that belongs to the interacting set. Similarly, a false positive (FP) PPI is defined as a predicted PPI that belongs to the non-interacting set. A true negative (TN) PPI prediction is defined as a predicted non-interacting protein pair that belongs to the non-interacting set. A false negative (FN) PPI prediction is defined as a predicted non-interacting protein pair that belongs to the interacting set. The true positive rate is then defined as TP/(TP + FN) and the false positive rate as FP/(FP + TN).

### Parameter-dependent DDI selection (PADDS) algorithm

PADDS was designed to select sets of DDIs that can reconstitute a given protein interaction network. Specifically, for each potential DDI and its corresponding PPIs, we assessed the consequences of selecting that particular DDI versus each one of the other possible DDIs that account for the same PPIs (we denoted these DDIs as alternative DDIs). Instead of exploring all possible combinations of alternative DDIs, PADDS explores only a subset of enumerations consisting of currently evaluated DDIs and their best alternatives, *i.e.*, alternatives that best satisfy the evaluation criteria. If the evaluated DDI was better than any alternative, it was selected as a PPI mediator and assigned a benefit score. Already selected DDIs, as well as the PPIs they accounted for, were never re-evaluated, further limiting the number of combinations to be enumerated. The final constructed set of domain interactions represented a minimal DDI set that accounted for all PPIs, while attempting to minimize false positives.

In contrast to existing reconstitution methods, PADDS does not *a priori* reward or penalize the most rare, promiscuous, or parsimonious set of interactions. Instead, it biases the benefit of each selected domain interaction towards either preferring observed PPIs (true positives) or penalizing non-observed PPIs (here categorized as false positives). Thus, depending on the value of the parameter that specifies the true/false positive biases (denoted as *α*; *α* ε [*0.0, 1.0*]), PADDS extracts multiple sets of potential DDIs that can explain the original set of PPIs. An *α* of 0.0 favors observed protein interactions and an *α* of 1.0 maximally penalizes non-observed interactions. In addition, PADDS also identifies a set of robust, core DDIs that are independent of the parameter *α*.

### Algorithm and implementation details

Let *O*_*ij*_ denote the number of observed interacting protein pairs, where one protein contains domain *i* and the other contains domain *j,* and let *N*_*ij*_ denote the number of all possible non-interacting protein pairs, where one protein contains domain *i* and the other contains domain *j*. The association score *A*_*ij*_[28], which represents the probability of interaction between domains *i* and *j*, is defined as:

(1) $$A_{\mathrm{ij}}=\frac{O_{\mathrm{ij}}}{O_{\mathrm{ij}}+N_{\mathrm{ij}}}.$$

Let *α* denote a parameter with a value in the [*0.0, 1.0*] range that specifies the amount of tolerable non-interacting protein pairs. We evaluated the probability of the occurrence of a domain pair *i* and *j* in a set of PPIs as a modified association score $A_{\mathrm{ij}}^{m}$:

(2) $$A_{\mathrm{ij}}^{m}=\frac{O_{\mathrm{ij}}{}^{2}}{O_{\mathrm{ij}}+\alpha .N_{\mathrm{ij}}}.$$

For *α = 0.0*, $A_{\mathrm{ij}}^{m}$ equals the number of observed DDIs, *i.e.*, the number of PPIs in which one protein contains domain *i* and the other contains domain *j*, whereas for *α = 1.0*, $A_{\mathrm{ij}}^{m}$ denotes the probability of interaction between domains *i* and *j* [defined in Equation (1)] multiplied by the number of domain interaction occurrences. Thus, for *α = 0.0*, the modified association score corresponds to domain interactions that explain the largest number of PPIs, while for *α = 1.0*, the score corresponds to domain interactions that do not introduce large number of false positive PPIs. We multiplied the probability of interaction by *O*_*ij*_ to differentiate between DDIs that have the same probability by assigning a higher score to those DDIs that account for a larger number of PPIs.

### Benefit definition

The benefit of interaction between two domains *i* and *j* represents the propensity that these two domains mediate protein interactions. We defined the benefit by combining the above modified association score with a term that takes into account the co-occurrence of domains, because domains that appear together within a protein often interact [46,47]. Let *C*_*ij*_ denote the number of proteins in which domains *i* and *j* co-occur, and let *maxC*_*ij*_ denote the maximum number of co-occurring domains observed in a given set of proteins. We then defined the benefit *B*_*ij*_ of the interaction between two domains *i* and *j* as:

(3) $$B_{\mathrm{ij}}=\frac{O_{\mathrm{ij}}{}^{2}}{O_{\mathrm{ij}}+\alpha .N_{\mathrm{ij}}}+\frac{C_{\mathrm{ij}}{}^{2}}{\max C_{\mathrm{ij}}}.$$

Using different values of *α*, one can rank the same set of DDIs differently based on their *B*_*ij*_ value.

### Iterative evaluation of selected DDIs

**Figure 5:**
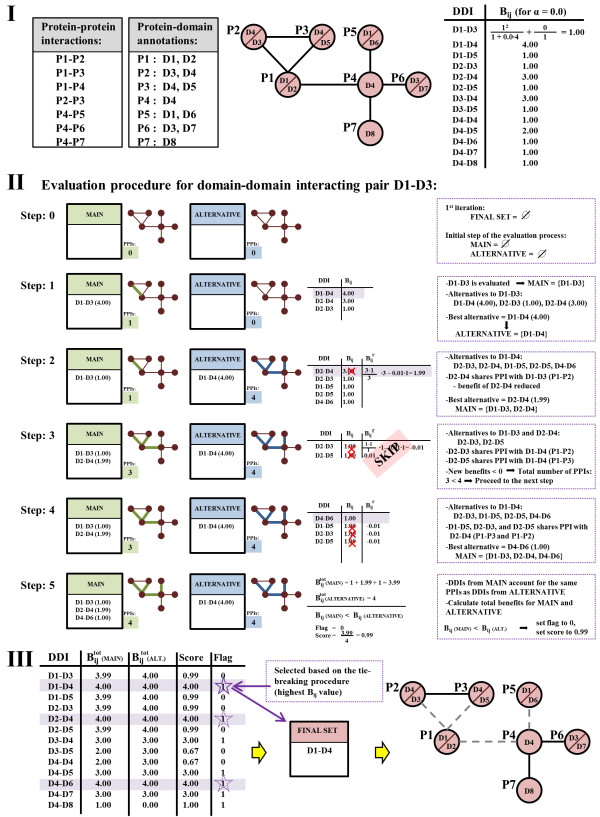
**Example of domain-domain interaction extraction. I**: Given a set of protein-protein interactions (PPIs) and a protein-domain annotation scheme, PADDS transformed all PPIs into the corresponding set of domain-domain interactions (DDIs) and calculated the benefit value *B*_*ij*_ for all DDIs. **II**: The five steps involved in the DDI iterative evaluation procedure is illustrated using interactions between domains D1 and D3. **III**: After PADDS performed the DDI evaluation procedure for all other DDIs, the results were examined to select the final set of DDIs that can reconstitute the PPIs. P1, …, P7 denote proteins and D1, …, D8 denote domains. The benefit *B*_*ij*_ and the reassessed benefit $B_{\mathrm{ij}}^{r}$ associated with the interaction between domains *ij* were calculated using Equations (3) and (4), respectively.

PADDS goal is to extract a set of DDIs such that: *1*) these DDIs account for a given set of PPIs and *2*) the sum of benefits of this set is higher than the sum of benefits of any alternative DDI set of the same size that explains the same PPIs. The optimal solution for this problem would require the exhaustive enumeration of all possible combinations of DDIs that account for the original set of PPIs. Because, in practice, the exhaustive enumeration is computationally unfeasible, PADDS uses a heuristic solution. Given a set of PPIs, a list of protein-domain annotations, and a user-specified parameter *α*, PADDS calculates *B*_*ij*_ for each potential DDI (Figure 5 – I). For each DDI and the corresponding PPIs represented by this DDI, PADDS evaluates the consequences of selecting this DDI versus each of its alternative DDIs. This evaluation yields a small set of DDIs, called the *final* set.

The evaluation process consists of five phases. In the first phase, PADDS adds a DDI of interest into a set called the *main* set. In addition, PADDS evaluates all alternative DDIs, *i.e.*, DDIs that overlap with the DDI of interest, and adds the one with the highest benefit to a set called the *alternative* set. Note that the *main* and *alternative* sets are initially empty (Figure 5 – II, Step: 0 and Step: 1). In the second phase, PADDS evaluates all DDIs that overlap with the DDIs from the *alternative* set (Figure 5 – II, Step: 2 to Step: 4). PADDS always evaluates only the DDIs that are not already contained in the *alternative, main,* or *final* sets. However, in the evaluation process, PADDS takes into consideration that DDIs from the *final* set already account for a particular subset of PPIs. Because these PPIs should not be used for the evaluation of potential DDIs, for all potential DDIs that are not in the *final* set, PADDS calculates the reassessed benefits $B_{\mathrm{ij}}^{r}$ as:

(4) $$B_{\mathrm{ij}}^{r}=\frac{O_{\mathrm{ij}}-E_{\mathrm{ij}}}{O_{\mathrm{ij}}}.B_{\mathrm{ij}}-s.E_{\mathrm{ij}},$$

where *E*_*ij*_ represents the number of observed interacting protein pairs containing domains *i* and *j* that have already been accounted for by some other DDI (either from the *main/alternative* set or the *final* set), and *s* represents a scaling factor used to additionally reduce the benefit value of DDIs. We empirically selected *s = 0.01.* Out of all evaluated DDIs, PADDS finds a DDI with the highest reassessed benefit and adds it to the *main* set. In the second phase, the algorithm iterates between the *alternative* set and the *main* set until all PPIs that are explained by potential DDIs in one set are also explained by potential DDIs from the other set. In phase three, PADDS calculates the total accumulative benefit $B_{\mathrm{ij}}^{\mathrm{tot}}$ for each set as:

(5) $$B_{\mathrm{ij}}^{\mathrm{tot}}=k.\underset{m,n}{\sum }B_{\mathrm{mn}}+\left(1-k\right).\underset{m,n}{\sum }B_{\mathrm{mn}}^{r},\;k=0\;\mathrm{or}\;1$$

where *k =* 1 for DDIs that were added into a set based on their original benefit value *B*_*mn*_, and *k =* 0 for DDIs that were added into a set based on their reassessed benefit value $B_{\mathrm{mn}}^{r}$ (Figure 5 – II, Step: 5). In the fourth phase, PADDS compares the $B_{\mathrm{ij}}^{\mathrm{tot}}$ value of the *main* and *alternative* sets. If the *main* set has the greatest $B_{\mathrm{ij}}^{\mathrm{tot}}$ value, PADDS flags the DDI of interest and assigns it this value. Then, in phase five, for all flagged DDIs, PADDS finds the one with the highest $B_{\mathrm{ij}}^{\mathrm{tot}}$ value and adds this DDI to the *final* set of DDIs (Figure 5 – II, Step: 5). In the case where no DDI is flagged, *i.e.*, all *alternative* sets have higher $B_{\mathrm{ij}}^{\mathrm{tot}}$ values than their corresponding *main* set counterparts, PADDS assigns to each DDI a value equal to the ratio of $B_{\mathrm{ij}}^{\mathrm{tot}}$ of the *main* set and $B_{\mathrm{ij}}^{\mathrm{tot}}$ of the *alternative* set. Then, PADDS adds DDI with the highest ratio value to the *final* set (Figure 5 – III). This evaluation procedure is repeated until all given PPIs are explained by DDIs from the *final* set. Extracted sets of potential DDIs that are common to all values of *α* are denoted as the *core* set.

Ties between two DDIs are broken in the following order: *1*) minimum *N*_*ij*_, *2*) maximum *O*_*ij*_, *3*) maximum number of times the interaction between domains *i* and *j* explains a single PPI multiple times (*e.g.*, if both proteins contain domains *i* and *j*, then that PPI can be explained by two *i* – *j* domain interactions), *4*) maximum *C*_*ij*_, *5*) maximum number of unique PPIs that a DDI explains, and *6*) maximum benefit. In cases where ties are not broken after this procedure, they are broken randomly.

### Data and implementation of other reconstitution methods: GPE and MSSC

For the comparison with the GPE method [21] on the dataset from Riley *et al*. [27], we used two sets of published results; the first contained the top 1,399 high-confidence DDIs (denoted “*GPE-HC*”) and the second contained 7,554 DDIs of lower-confidence (denoted “*GPE-LC*”) that were not necessarily included in the first set. In the comparisons, we used the DDI rank information provided with the published data [21].

For the yeast high-confidence dataset comparison, we used the MSSC program available from the authors’ Web site to extract and rank DDIs using the association score [28]. We implemented the GPE algorithm in MATLAB using the parameter values specified by the authors and ranked DDIs using the LP-score, following the methodology detailed in the original manuscript [21].

## Endnotes

^a^This set of proteins contains the translations of all systematically named ORFs, except ORFs designated as “dubious” or “pseudogenes.”

^b^The remaining domain annotations did not change, had minor modifications compared to the previous version, had domains of unknown function, had domains assigned to previously unannotated proteins, or had domains assigned to previously unannotated sequence segments.

## Abbreviations

CDD: Conserved Domain Database; DDI: Domain-domain interaction; FN: False negative; FP: False positive; GPE: Generalized parsimonious explanation; IDBOS: Interaction detection based on shuffling; MSSC: Maximum-specificity set cover; PA: Parsimonious approach; PADDS: Parameter-dependent DDI selection; PPI: Protein-protein interaction; ROC: Receiver operating characteristic; SF: Superfamily; SGD: Saccharomyces Genome Database; TN: True negative; TP: True positive.

## Competing interests

The authors declare that they have no competing interests.

## Authors’ contributions

VM, AW, and JR conceived of the study and participated in its design and coordination. VM collected the data, developed the algorithms, and performed the calculations. VM and AW drafted the original manuscript, which was edited by JR. All authors read and approved the final manuscript.

## Supplementary Material

Additional file 1 **Merged annotation.** This file contains lists of new protein-domain annotations and lists that map new domain labels onto the domain labels used in the original annotation databases. Click here for file

Additional file 2 **Merged protein-domain annotations that correspond to domain annotations from the new PFAM-A release.** This file contains a list of 202 merged protein-domain annotations corresponding to domain annotations from the current PFAM-A release (release 26.0) that did not exist in the previous PFAM-A release (release 25.0), but had a matching domain annotation in our merged set. Additionally, this file contains the merged protein-domain annotations corresponding to the current PFAM-A domain annotations that replaced the annotations from the previous PFAM release. Click here for file

Additional file 3 **Domain-domain interactions extracted by the *parameter-dependent DDI selection* (PADDS) method – multiple organisms.** This file contains lists of domain-domain interactions extracted by PADDS for the Riley dataset [27] for all values of the parameter *α* analyzed in the main text. Click here for file

Additional file 4 **Supplementary material.** This file provides the supplemental text, supplemental Additional file 4: Figures S1 – S4, and supplemental Additional file 4: Tables S1 – S4. Click here for file

## References

[B1] Hart GT Ramani AK Marcotte EM How complete are current yeast and human protein-interaction networks? Genome Biol 2006 7 11 120 10.1186/gb-2006-7-11-120 17147767 PMC1794583

[B2] Sambourg L Thierry-Mieg N New insights into protein-protein interaction data lead to increased estimates of the *S. cerevisiae* interactome size BMC Bioinformatics 2010 11 605 10.1186/1471-2105-11-605 21176124 PMC3023808

[B3] Stumpf MP Thorne T de Silva E Stewart R An HJ Lappe M Wiuf C Estimating the size of the human interactome Proc Natl Acad Sci USA 2008 105 19 6959 6964 10.1073/pnas.0708078105 18474861 PMC2383957

[B4] von Mering C Krause R Snel B Cornell M Oliver SG Fields S Bork P Comparative assessment of large-scale data sets of protein-protein interactions Nature 2002 417 6887 399 403 12000970 10.1038/nature750

[B5] Yu H Braun P Yildirim MA Lemmens I Venkatesan K Sahalie J Hirozane-Kishikawa T Gebreab F Li N Simonis N High-quality binary protein interaction map of the yeast interactome network Science 2008 322 5898 104 110 10.1126/science.1158684 18719252 PMC2746753

[B6] Cusick ME Yu H Smolyar A Venkatesan K Carvunis AR Simonis N Rual JF Borick H Braun P Dreze M Literature-curated protein interaction datasets Nat Methods 2009 6 1 39 46 10.1038/nmeth.1284 19116613 PMC2683745

[B7] Venkatesan K Rual JF Vazquez A Stelzl U Lemmens I Hirozane-Kishikawa T Hao T Zenkner M Xin X Goh KI An empirical framework for binary interactome mapping Nat Methods 2009 6 1 83 90 10.1038/nmeth.1280 19060904 PMC2872561

[B8] Yu X Ivanic J Memišević V Wallqvist A Reifman J Categorizing biases in high-confidence high-throughput protein-protein interaction data sets Mol Cell Proteomics 2011 10 12 M111 012500 21876202 10.1074/mcp.M111.012500 PMC3237088

[B9] Yu X Wallqvist A Reifman J Inferring high-confidence human protein-protein interactions BMC Bioinformatics 2012 13 79 10.1186/1471-2105-13-79 22558947 PMC3416704

[B10] Apic G Gough J Teichmann SA Domain combinations in archaeal, eubacterial and eukaryotic proteomes J Mol Biol 2001 310 2 311 325 10.1006/jmbi.2001.4776 11428892

[B11] Gupta S Wallqvist A Bondugula R Ivanic J Reifman J Unraveling the conundrum of seemingly discordant protein-protein interaction datasets Conf Proc IEEE Eng Med Biol Soc 2010 2010 783 786 21096109 10.1109/IEMBS.2010.5626490

[B12] Itzhaki Z Akiva E Altuvia Y Margalit H Evolutionary conservation of domain-domain interactions Genome Biol 2006 7 12 R125 10.1186/gb-2006-7-12-r125 17184549 PMC1794438

[B13] Ekman D Björklund AK Frey-Skött J Elofsson A Multi-domain proteins in the three kingdoms of life: orphan domains and other unassigned regions J Mol Biol 2005 348 1 231 243 10.1016/j.jmb.2005.02.007 15808866

[B14] Yang S Bourne PE The evolutionary history of protein domains viewed by species phylogeny PLOS ONE 2009 4 12 e8378 10.1371/journal.pone.0008378 20041107 PMC2794708

[B15] Marchler-Bauer A Lu S Anderson JB Chitsaz F Derbyshire MK DeWeese-Scott C Fong JH Geer LY Geer RC Gonzales NR CDD: a Conserved Domain Database for the functional annotation of proteins Nucleic Acids Res 2011 39 Database issue D225 D229 21109532 10.1093/nar/gkq1189 PMC3013737

[B16] Apweiler R Attwood TK Bairoch A Bateman A Birney E Biswas M Bucher P Cerutti L Corpet F Croning MD InterPro–an integrated documentation resource for protein families, domains and functional sites Bioinformatics 2000 16 12 1145 1150 10.1093/bioinformatics/16.12.1145 11159333

[B17] Hunter S Apweiler R Attwood TK Bairoch A Bateman A Binns D Bork P Das U Daugherty L Duquenne L InterPro: the integrative protein signature database Nucleic Acids Res 2009 37 Database issue D211 D215 18940856 10.1093/nar/gkn785 PMC2686546

[B18] Chen XW Liu M Prediction of protein-protein interactions using random decision forest framework Bioinformatics 2005 21 24 4394 4400 10.1093/bioinformatics/bti721 16234318

[B19] Deng M Mehta S Sun F Chen T Inferring domain-domain interactions from protein-protein interactions Genome Res 2002 12 10 1540 1548 10.1101/gr.153002 12368246 PMC187530

[B20] Guimarães KS Jothi R Zotenko E Przytycka TM Predicting domain-domain interactions using a parsimony approach Genome Biol 2006 7 11 R104 10.1186/gb-2006-7-11-r104 17094802 PMC1794579

[B21] Guimarães KS Przytycka TM Interrogating domain-domain interactions with parsimony based approaches BMC Bioinformatics 2008 9 171 10.1186/1471-2105-9-171 18366803 PMC2358894

[B22] Hayashida M Ueda N Akutsu T A simple method for inferring strengths of protein-protein interactions Genome Inform 2004 15 1 56 68 15712110

[B23] Huang C Morcos F Kanaan SP Wuchty S Chen DZ Izaguirre JA Predicting protein-protein interactions from protein domains using a set cover approach IEEE/ACM Trans Comput Biol Bioinform 2007 4 1 78 87 17277415 10.1109/TCBB.2007.1001

[B24] Lee H Deng M Sun F Chen T An integrated approach to the prediction of domain-domain interactions BMC Bioinformatics 2006 7 269 10.1186/1471-2105-7-269 16725050 PMC1481624

[B25] Liu M Chen XW Jothi R Knowledge-guided inference of domain-domain interactions from incomplete protein-protein interaction networks Bioinformatics 2009 25 19 2492 2499 10.1093/bioinformatics/btp480 19667081 PMC2752622

[B26] Nye TM Berzuini C Gilks WR Babu MM Teichmann SA Statistical analysis of domains in interacting protein pairs Bioinformatics 2005 21 7 993 1001 10.1093/bioinformatics/bti086 15509600

[B27] Riley R Lee C Sabatti C Eisenberg D Inferring protein domain interactions from databases of interacting proteins Genome Biol 2005 6 10 R89 10.1186/gb-2005-6-10-r89 16207360 PMC1257472

[B28] Sprinzak E Margalit H Correlated sequence-signatures as markers of protein-protein interaction J Mol Biol 2001 311 4 681 692 10.1006/jmbi.2001.4920 11518523

[B29] Yip KY Kim PM McDermott D Gerstein M Multi-level learning: improving the prediction of protein, domain and residue interactions by allowing information flow between levels BMC Bioinformatics 2009 10 241 10.1186/1471-2105-10-241 19656385 PMC2734556

[B30] Zhao XM Chen L Aihara K A discriminative approach for identifying domain-domain interactions from protein-protein interactions Proteins 2010 78 5 1243 1253 10.1002/prot.22643 20027642

[B31] Cherry JM Ball C Weng S Juvik G Schmidt R Adler C Dunn B Dwight S Riles L Mortimer RK Genetic and physical maps of *Saccharomyces cerevisiae* Nature 1997 387 6632 Suppl 67 73 9169866 PMC3057085

[B32] Finn RD Mistry J Tate J Coggill P Heger A Pollington JE Gavin OL Gunasekaran P Ceric G Forslund K The Pfam protein families database Nucleic Acids Res 2010 38 Database issue D211 D222 19920124 10.1093/nar/gkp985 PMC2808889

[B33] Gough J Karplus K Hughey R Chothia C Assignment of homology to genome sequences using a library of hidden Markov models that represent all proteins of known structure J Mol Biol 2001 313 4 903 919 10.1006/jmbi.2001.5080 11697912

[B34] Schultz J Milpetz F Bork P Ponting CP SMART, a simple modular architecture research tool: identification of signaling domains Proc Natl Acad Sci USA 1998 95 11 5857 5864 10.1073/pnas.95.11.5857 9600884 PMC34487

[B35] Letunic I Doerks T Bork P SMART 6: recent updates and new developments Nucleic Acids Res 2009 37 Database issue D229 D232 18978020 10.1093/nar/gkn808 PMC2686533

[B36] Bru C Courcelle E Carrere S Beausse Y Dalmar S Kahn D The ProDom database of protein domain families: more emphasis on 3D Nucleic Acids Res 2005 33 Database issue D212 D215 15608179 10.1093/nar/gki034 PMC539988

[B37] Haft DH Loftus BJ Richardson DL Yang F Eisen JA Paulsen IT White O TIGRFAMs: a protein family resource for the functional identification of proteins Nucleic Acids Res 2001 29 1 41 43 10.1093/nar/29.1.41 11125044 PMC29844

[B38] Finn RD Marshall M Bateman A iPfam: visualization of protein-protein interactions in PDB at domain and amino acid resolutions Bioinformatics 2005 21 3 410 412 10.1093/bioinformatics/bti011 15353450

[B39] Raghavachari B Tasneem A Przytycka TM Jothi R DOMINE: a database of protein domain interactions Nucleic Acids Res 2008 36 Database issue D656 D661 17913741 10.1093/nar/gkm761 PMC2238965

[B40] Yellaboina S Tasneem A Zaykin DV Raghavachari B Jothi R DOMINE: a comprehensive collection of known and predicted domain-domain interactions Nucleic Acids Res 2011 39 Database issue D730 D735 21113022 10.1093/nar/gkq1229 PMC3013741

[B41] Yu X Ivanic J Wallqvist A Reifman J A novel scoring approach for protein co-purification data reveals high interaction specificity PLOS Comput Biol 2009 5 9 e1000515 10.1371/journal.pcbi.1000515 19779545 PMC2738424

[B42] Dobson CM Protein folding and misfolding Nature 2003 426 6968 884 890 10.1038/nature02261 14685248

[B43] Stein A Panjkovich A Aloy P 3did Update: domain-domain and peptide-mediated interactions of known 3D structure Nucleic Acids Res 2009 37 Database issue D300 D304 18953040 10.1093/nar/gkn690 PMC2686500

[B44] Stein A Russell RB Aloy P 3did: interacting protein domains of known three-dimensional structure Nucleic Acids Res 2005 33 Database issue D413 D417 15608228 10.1093/nar/gki037 PMC539991

[B45] Björklund AK Ekman D Elofsson A Expansion of protein domain repeats PLOS Comput Biol 2006 2 8 e114 10.1371/journal.pcbi.0020114 16933986 PMC1553488

[B46] Enright AJ Iliopoulos I Kyrpides NC Ouzounis CA Protein interaction maps for complete genomes based on gene fusion events Nature 1999 402 6757 86 90 10.1038/47056 10573422

[B47] Marcotte EM Pellegrini M Ng HL Rice DW Yeates TO Eisenberg D Detecting protein function and protein-protein interactions from genome sequences Science 1999 285 5428 751 753 10.1126/science.285.5428.751 10427000

