# Supplementary material for: Reconstituting protein interaction networks using parameter-dependent domain-domain interactions
Source: BMC Bioinformatics. 2013 May 7;14:154. doi: 10.1186/1471-2105-14-154 (PMC3660195; doi:10.1186/1471-2105-14-154)
Supplement: Additional file 4 — Supplementary material. This file provides the supplemental text, supplemental Additional file 4: Figures S1 – S4, and supplemental Additional file 4: Tables S1 – S4. [file 1471-2105-14-154-S4.pdf]

Supplementary material: Reconstituting protein interaction networks using parameter-dependent domain-domain interactions

Vesna Memišević, Anders Wallqvist, and Jaques Reifman[§]

Department of Defense Biotechnology High Performance Computing Software Applications Institute, Telemedicine and Advanced Technology Research Center, U.S. Army Medical Research and Materiel Command, Fort Detrick, MD 21702

[§]Corresponding author

E-mail addresses:

VM: vmemisevic@bhsai.org

AW: awallqvist@bhsai.org

JR: jaques.reifman@us.army.mil

Additional Text

Validation of extracted core DDIs

We used the DOMINE database as a comprehensive source of known and predicted domain-domain interactions (DDIs) derived from multiple sources [1, 2]. In the sets of DDIs extracted by the *parameter-dependent DDI selection* (PADDS) method for protein-protein interactions (PPIs) from the Riley dataset, 12,725 DDIs were conserved across all values of α and represented a core set of DDIs for this PPI set. We ranked the DDIs extracted by PADDS based on their corresponding benefit values and evaluated the k top-ranked DDIs. For each threshold k, k = *500, 1,500, 3,000, 5,000, 10,000, 15,000*, we identified a set of core DDIs, *i.e.*, DDIs that did not depend on α, that appeared in the top k DDIs in all extracted sets. We compared the core DDIs to those from the DOMINE database [1, 2] and identified the fractions that had already been *1*) extracted/predicted solely by other computational methods, *2*) derived from a crystal structure and extracted/predicted by other computational methods, or *3*) derived from a crystal structure and extracted by PADDS but not by any other computational method.

Figure S1 below shows the types and fractions of DOMINE-validated core DDIs for each set extracted at different thresholds of top-ranked DDIs inferred by PADDS. As expected, increasing the threshold for selecting top-scoring DDIs decreased the overall percentage of validated DDIs in the core sets. However, even for the complete core set of recovered DDIs, we were still able to validate approximately 40% of the core DDIs in the DOMINE database.

Among the top-ranked DDIs for thresholds ≤1,500, the extracted core sets were enriched with interactions derived from known structures (shown in red and green in Figure S1). Out of the 220 top core DDIs, 122 were inferred from Protein Data Bank

entries [3-5]. Five of these 122 DDIs had not been detected by any other computational method, namely interactions between: *1*) helicase conserved C-terminal domain (annotation label: *HELICASE_C*) and type III restriction enzyme (*RESIII*), *2*) protein kinase domain (*PKINASE*) and immunoglobulin I-set domain (*I-SET*), *3*) pyrroline-5-carboxylate reductase domains (*P5CR*), *4*) bZIP Maf transcription factors (*BZIP MAF*), and *5*) Src homology 3 domains *SH3_1* and *SH3_2*. Thus, the PADDS algorithm was capable of providing parameter-independent and unique DDI predictions not derivable from high-confidence results of other computational procedures.

Additional Figures

Figure S1 – Enrichment in DOMINE domain-domain interactions

For each threshold *k*, the number of core domain-domain interactions (DDIs) identified by the *parameter-dependent DDI selection* (PADDS) method is shown on the x-axis and the corresponding *k*-value given underneath in parenthesis. We compared the core DDIs to those from the DOMINE database [1, 2] and identified the fractions that had already been *1*) extracted/predicted solely by other computational methods (yellow), *2*) derived from a crystal structure and extracted/predicted by other computational methods (red), or *3*) derived from a crystal structure and extracted by PADDS but not by any other computational method (green).

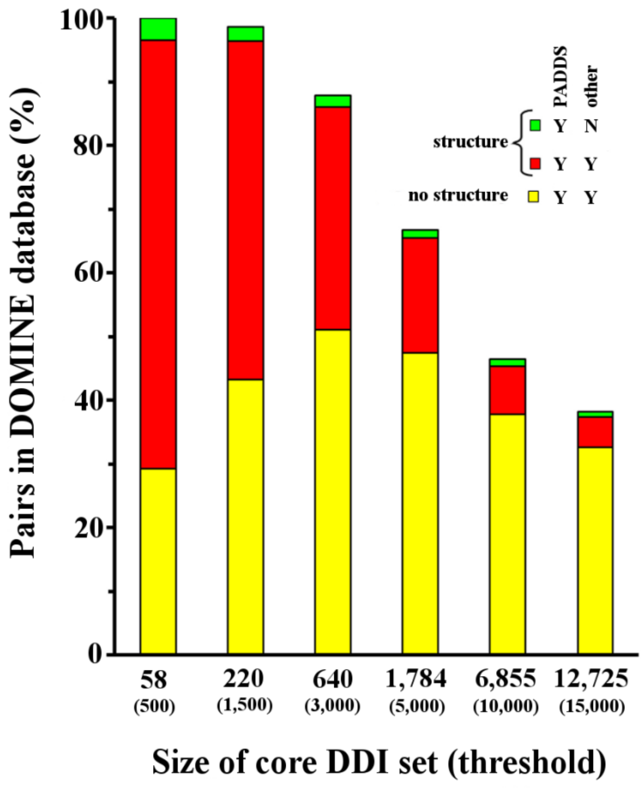

Figure S2 – Relationship between the amount of domain annotation data, the number of extracted domain-domain interactions, and the number of predicted protein-protein interactions for yeast

Three different reconstitution methods {the *maximum-specificity set cover* method (MSSC) [7], the *generalized parsimonious explanation* (GPE) [8], and the *parameter-dependent DDI selection* (PADDS)} extracted domain-domain interaction (DDI) sets of different sizes, when we used six domain annotation sets containing data from different sources. Database sets were defined as in Table 2 of the main text. The reported PADDS values correspond to the average values over all extracted sets, *i.e.*, sets for all values of parameter α used, for the particular domain annotation set. PADDS consistently produced the smallest sets of extracted DDIs needed to account for a given set of protein-protein interactions (PPIs) and the size of these sets decreased with additional annotation data. The MSSC method extracted smaller sets of DDIs than GPE, for the first five sets of domain annotations. However, for the annotation set that contained domain annotation data from the six databases, MSSC extracted slightly larger sets of DDIs than GPE. All three methods yielded much larger numbers of possible PPIs for a given set of DDIs than the total estimated true number of yeast PPIs [16-19]. The marker size and the number corresponds to the number of merged databases, *e.g.*, 1 corresponds to SET-1, 2 corresponds to SET-2, etc. As the underlying set of PPIs, we used a high-confidence yeast PPI data set created by the Interaction Detection Based On Shuffling (IDBOS) procedure at a 5% false discovery rate [20, 21].

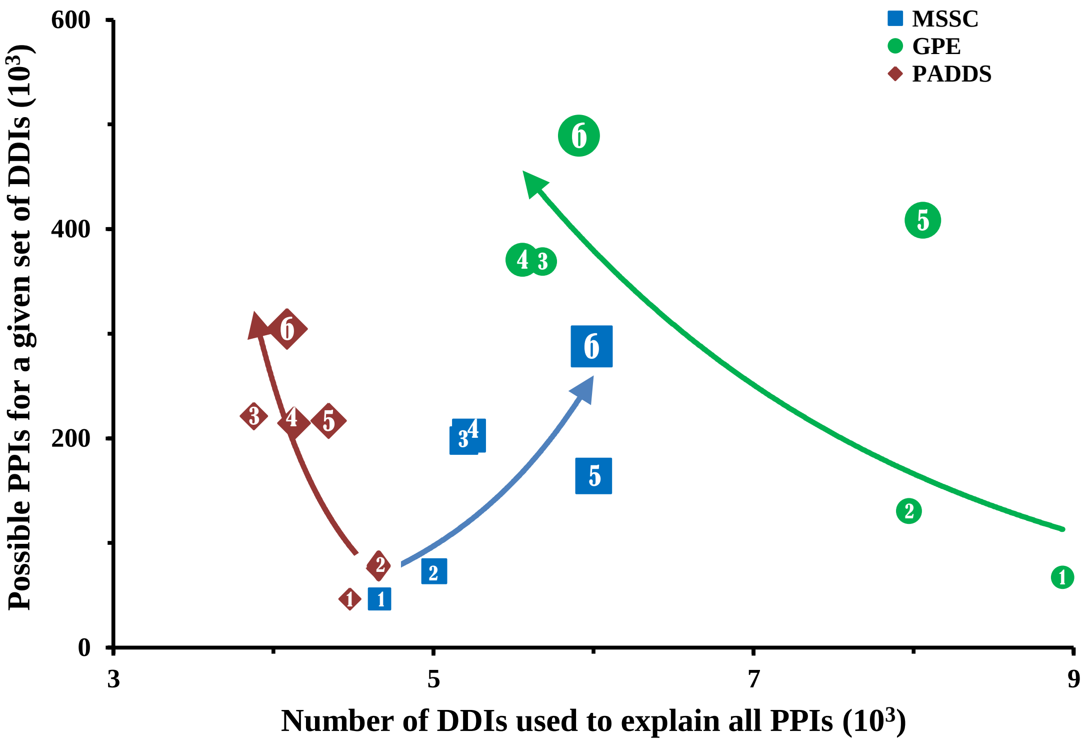

MSSC
GPE
PADDS
600
400
200
0
3
5
7
9
Possible PPIs for a given set of DDIs (10³)
Number of DDIs used to explain all PPIs (10³)
1
2
3
4
5
6

Figure S3 – Evaluation of reconstitution methods using receiver operating characteristic curve analysis

Comparison of the ability of each reconstitution method to extract domain-domain interactions (DDIs) that account for the underlying protein-protein interactions (PPIs) and novel PPIs. The true positive rate (true positive PPIs/(true positive PPIs + false negative PPIs) and the false positive rate (false positive PPIs/(false positive PPIs + true negative PPIs) for each extracted set of DDIs are represented as corresponding receiver operating characteristic curves. To estimate true/false negatives, we assumed that the set of negatives included all possible PPIs that were not in a given set of PPIs. We ranked DDIs based on benefit [the *parameter-dependent DDI selection* method (PADDS)], association score {the *maximum-specificity set cover* method (MSSC) [7]}, and LC score {the *generalized parsimonious explanation* (GPE) [8]}. We only plotted PADDS results for three values of α: *0.0, 0.1,* and *1.0*. Results for $\alpha \epsilon$ [*0.2, 0.9*] were equally distributed between the results for $\alpha = 0.1$ and $\alpha = 1.0$. PADDS for $\alpha > 0.0$ outperformed the MSSC and GPE methods. Although all methods (and parameters) produced very similar results, with increasing amounts of annotation data the differentiation between extracted DDIs and, hence, the methods and the parameters became more distinct. Database sets were defined as in Table 2 of the main text. For the complete data representation, see Figure S3. As the underlying set of PPIs, we used a high-confidence yeast PPI data set created by the Interaction Detection Based On Shuffling (IDBOS) procedure at a 5% false discovery rate [20, 21].

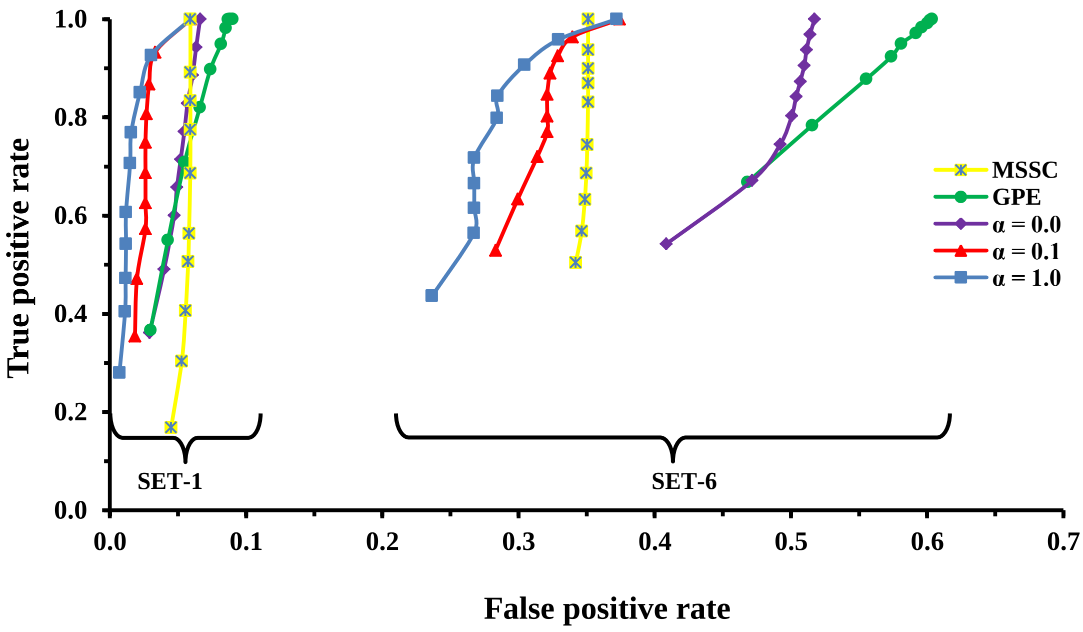

True positive rate
False positive rate
SET-1
SET-6
MSSC
GPE
α = 0.0
α = 0.1
α = 1.0
0.0
0.2
0.4
0.6
0.8
1.0
0.0
0.1
0.2
0.3
0.4
0.5
0.6
0.7

Figure S4 – Evaluation of reconstitution methods using receiver operating characteristic curve analysis: complete data

Comparison of the ability of each reconstitution method to extract domain-domain interactions (DDIs) that account for the underlying protein-protein interactions (PPIs) and novel PPIs. The true positive rate (true positive PPIs/(true positive PPIs + false negative PPIs) and the false positive rate (false positive PPIs/(false positive PPIs + true negative PPIs) for each extracted set of DDIs are represented as corresponding receiver operating characteristic curves. To estimate true/false negatives, we assumed that the set of negatives included all possible PPIs that were not in a given set of PPIs. We ranked DDIs based on benefit [the *parameter-dependent DDI selection* method (PADDS)], association score {the *maximum-specificity set cover* method (MSSC) [7]}, and LC score {the *generalized parsimonious explanation* (GPE) [8]}. We only plotted PADDS results for three values of α: *0.0, 0.1,* and *1.0*. Results for $\alpha \in [0.2, 0.9]$ were equally distributed between the results for $\alpha = 0.1$ and $\alpha = 1.0$. PADDS for $\alpha > 0.0$ outperformed the MSSC and GPE methods. Although all methods (and parameters) produced very similar results, with increasing amounts of annotation data the differentiation between extracted DDIs and, hence, the methods and the parameters became more distinct. Database sets were defined as in Table 2 of the main text. As the underlying set of PPIs, we used a high-confidence yeast PPI data set created by the Interaction Detection Based On Shuffling (IDBOS) procedure at a 5% false discovery rate [20, 21].

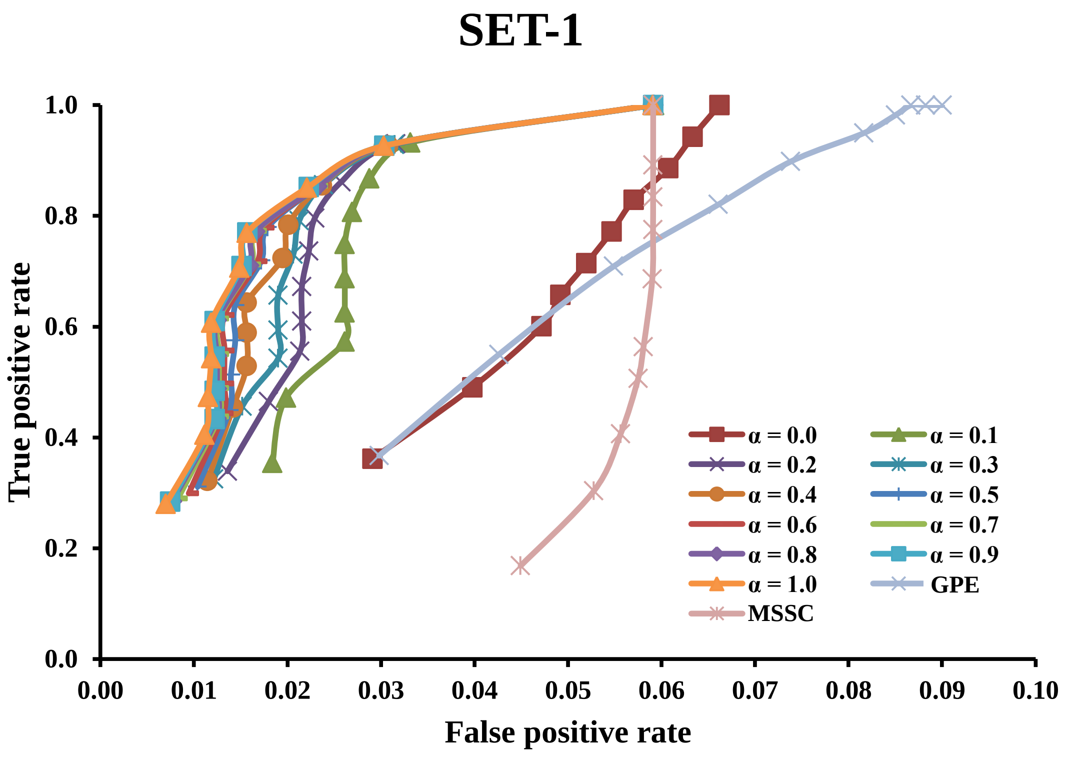
SET-1
True positive rate
1.0
0.8
0.6
0.4
0.2
0.0
0.00
0.01
0.02
0.03
0.04
0.05
0.06
0.07
0.08
0.09
0.10
False positive rate
α = 0.0
α = 0.1
α = 0.2
α = 0.3
α = 0.4
α = 0.5
α = 0.6
α = 0.7
α = 0.8
α = 0.9
α = 1.0
GPE
MSSC

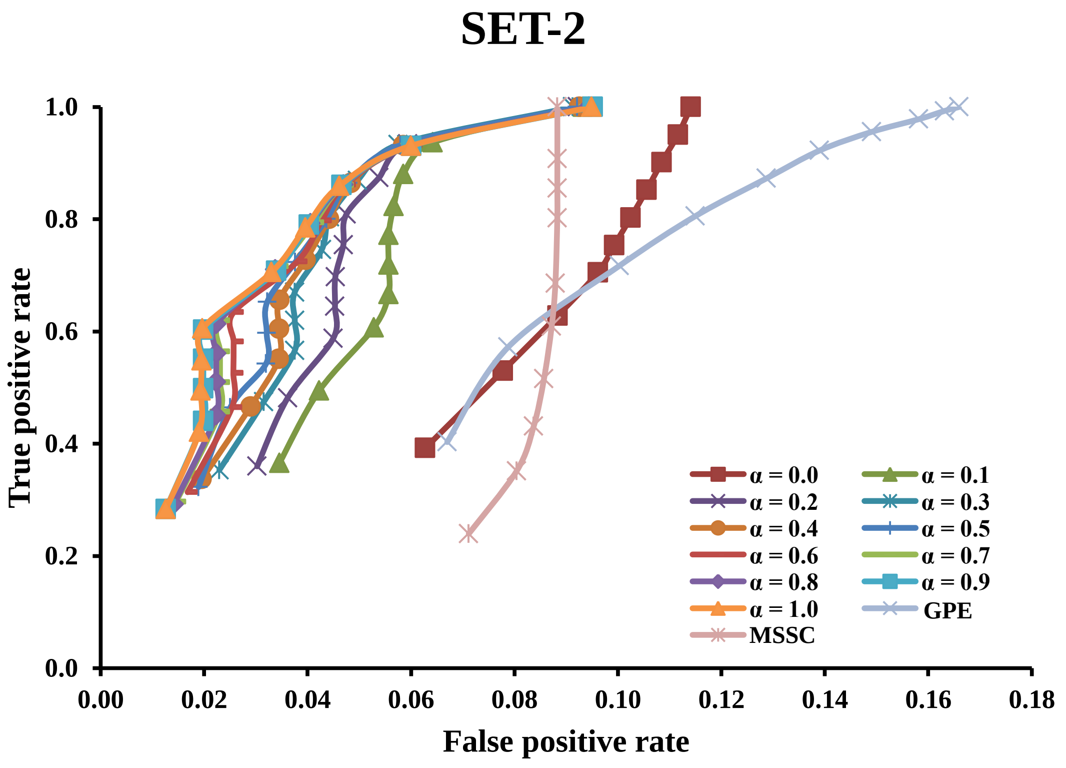
SET-2
True positive rate
1.0
0.8
0.6
0.4
0.2
0.0
0.00
0.02
0.04
0.06
0.08
0.10
0.12
0.14
0.16
0.18
False positive rate
α = 0.0
α = 0.1
α = 0.2
α = 0.3
α = 0.4
α = 0.5
α = 0.6
α = 0.7
α = 0.8
α = 0.9
α = 1.0
GPE
MSSC

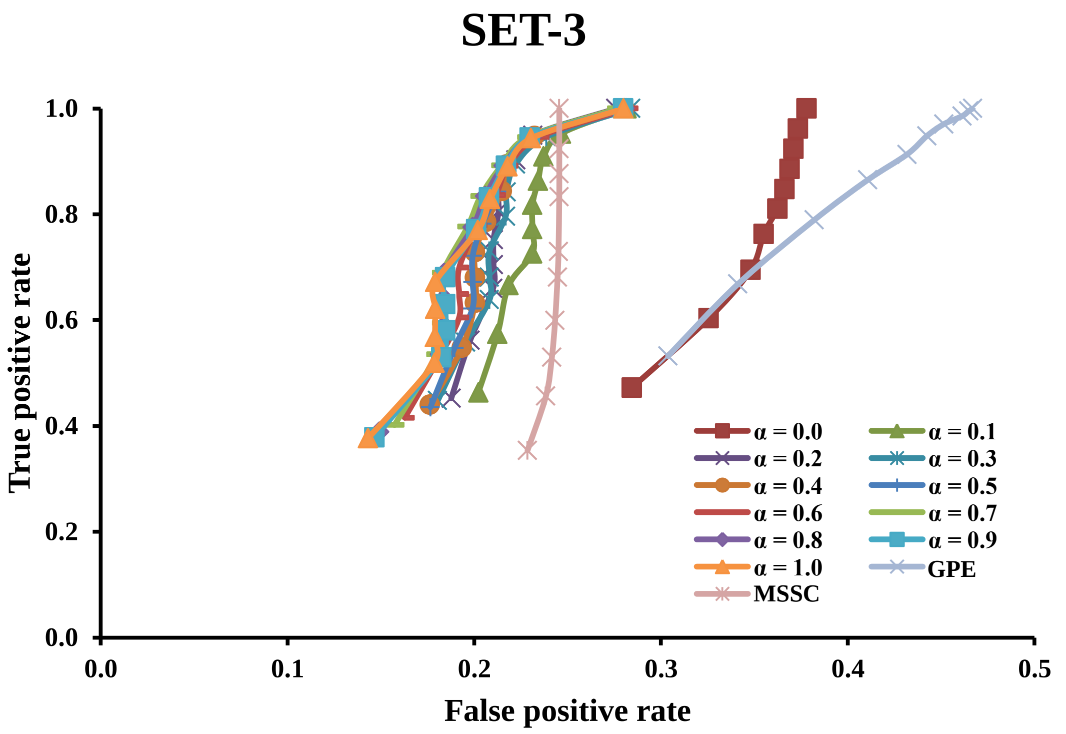
SET-3
True positive rate
False positive rate
1.0
0.8
0.6
0.4
0.2
0.0
0.0
0.1
0.2
0.3
0.4
0.5
α = 0.0
α = 0.1
α = 0.2
α = 0.3
α = 0.4
α = 0.5
α = 0.6
α = 0.7
α = 0.8
α = 0.9
α = 1.0
GPE
MSSC

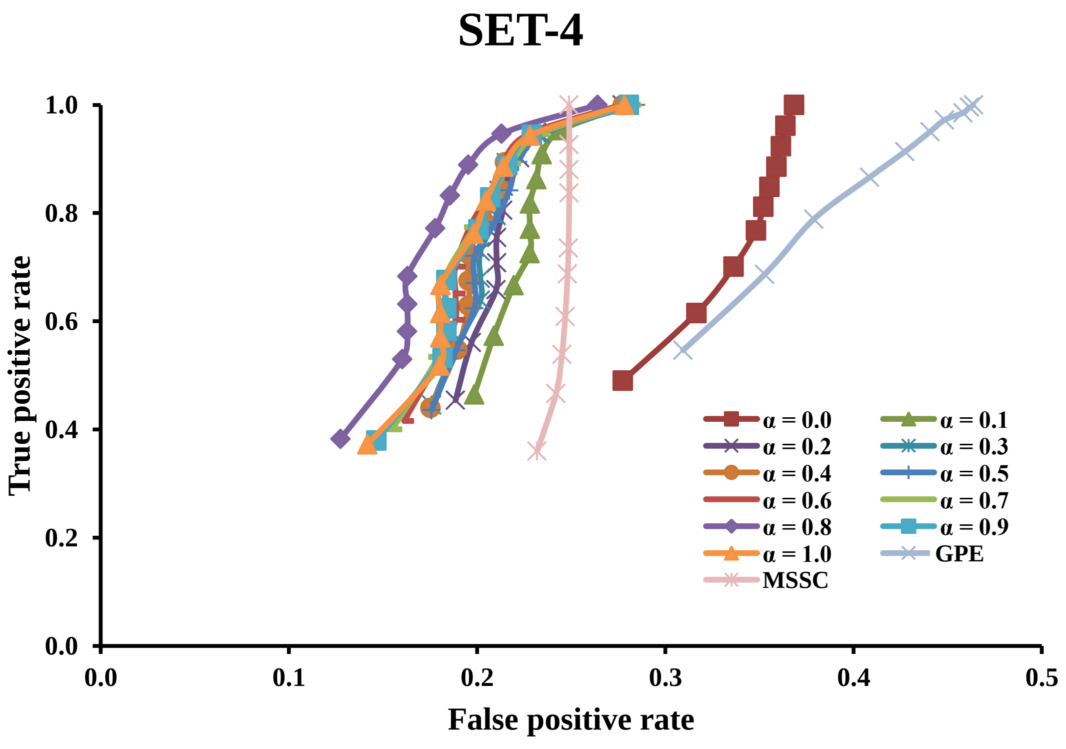
SET-4
True positive rate
False positive rate
1.0
0.8
0.6
0.4
0.2
0.0
0.0
0.1
0.2
0.3
0.4
0.5
α = 0.0
α = 0.1
α = 0.2
α = 0.3
α = 0.4
α = 0.5
α = 0.6
α = 0.7
α = 0.8
α = 0.9
α = 1.0
GPE
MSSC

SET-5

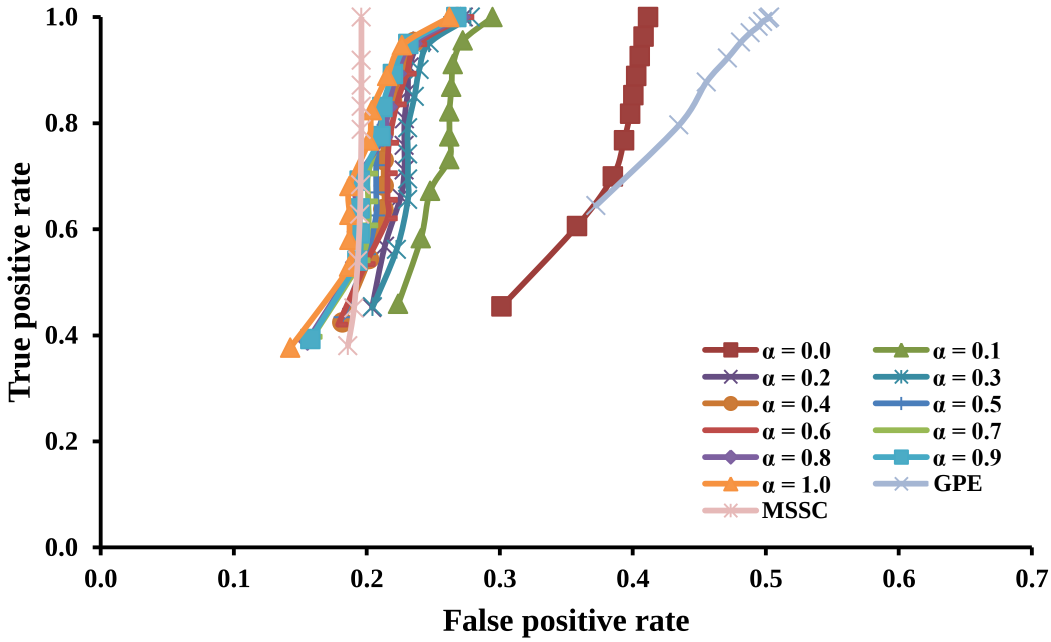

1.0
0.8
0.6
0.4
0.2
0.0
True positive rate
0.0
0.1
0.2
0.3
0.4
0.5
0.6
0.7
False positive rate
α = 0.0
α = 0.1
α = 0.2
α = 0.3
α = 0.4
α = 0.5
α = 0.6
α = 0.7
α = 0.8
α = 0.9
α = 1.0
GPE
MSSC

SET-6

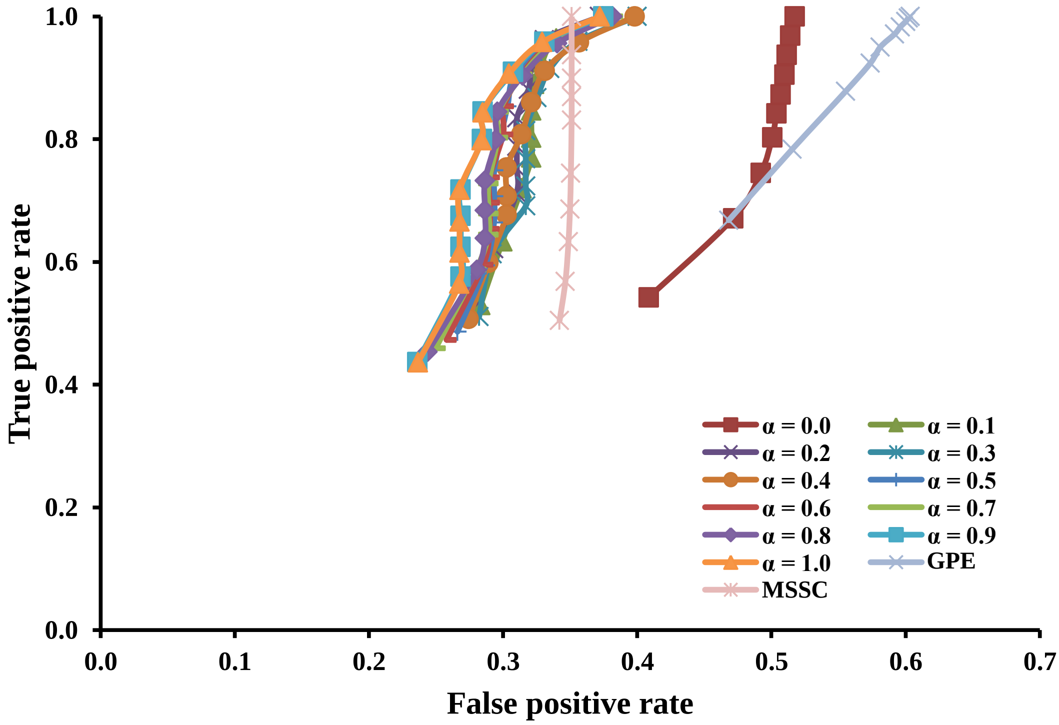

1.0
0.8
0.6
0.4
0.2
0.0
True positive rate
0.0
0.1
0.2
0.3
0.4
0.5
0.6
0.7
False positive rate
α = 0.0
α = 0.1
α = 0.2
α = 0.3
α = 0.4
α = 0.5
α = 0.6
α = 0.7
α = 0.8
α = 0.9
α = 1.0
GPE
MSSC

Tables

Table S1 – Enrichment of "known" (iPFAM) domain-domain interactions. Comparison of the fraction of retrieved iPFAM DDIs using PADDS and GPE as a function of top-ranked DDI sets. For the GPE sets, we used the DDI rank information provided with the published data that includes their designated high-confidence (GPE-HC) and low-confidence (GPE-LC) sets [8]. We have also indicated the best results achievable with any α value, typically achieved for ($\alpha = 0.1$). Data presented in this table correspond to the data from the Figure 2 of the main text. σ, standard deviation.

	DDI set size	PADDS-best	Non-extreme α values (σ)	GPE-HC	GPE-LC
Known DDIs retrieved (%)	**10**	1.01	0.77 (0.10)	1.45	0.72
	**50**	3.62	3.28 (0.22)	2.30	2.60
	**100**	7.53	6.82 (0.68)	5.21	4.78
	**250**	16.35	15.82 (0.49)	13.89	8.54
	**500**	27.21	25.74 (0.95)	22.14	15.63
	**1,000**	40.23	36.18 (1.75)	31.84	25.76
	**1,399**	43.56	38.75 (1.36)	34.59	30.97
	**2,000**	46.16	45.01 (0.69)	-	38.93
	**3,000**	51.66	51.15 (0.36)	-	46.02
	**5,000**	58.90	58.37 (0.31)	-	54.55
Precision	**10**	0.70	0.53 (0.07)	1.00	0.50
	**50**	0.50	0.45 (0.03)	0.32	0.36
	**100**	0.52	0.47 (0.05)	0.36	0.33
	**250**	0.45	0.44 (0.01)	0.38	0.24
	**500**	0.38	0.36 (0.01)	0.31	0.22
	**1,000**	0.28	0.25 (0.01)	0.22	0.18
	**1,399**	0.22	0.20 (0.01)	0.17	0.15
	**2,000**	0.16	0.16 (0.00)	-	0.13
	**3,000**	0.12	0.12 (0.00)	-	0.11
	**5,000**	0.08	0.08 (0.00)	-	0.08

Table S2 – Protein-domain annotation data for the IDBOS set of protein-protein interactions.

Domain annotation sets include merged domain annotation data from multiple databases and are defined as in Table 2 of the main text. The IDBOS data consisted of 1,295 proteins and 8,401 protein-protein interactions (PPIs) [20, 21]. The number and percentage of PPIs in which both interacting proteins have domain annotations are shown in columns 4 and 5, respectively [20, 21].

Domain annotation set	Yeast proteins in IDBOS set with domain annotation		PPIs with domain annotation		Average number of domains per yeast protein in the IDBOS set
	Number	Percentage	Number	Percentage	
SET-1	1,157	89.3	6,996	83.3	1.29
SET-2	1,217	94.0	7,766	92.4	1.63
SET-3	1,244	96.1	8,003	95.3	1.93
SET-4	1,251	96.6	8,044	95.8	1.91
SET-5	1,262	97.5	8,122	96.7	2.94
SET-6	1,263	97.5	8,138	96.9	2.69

Table S3 – Basic statistics of the extracted sets of domain-domain interactions using different methods and different protein-domain annotation sets.

Sets are defined as in Table 2 of the main text. Methods used to extract DDIs: the *parameter-dependent DDI selection* (PADDS), *maximum-specificity set cover* method (MSSC) [7], and the *generalized parsimonious explanation* (GPE) [8]. "ALL POSSIBLE DDIs" represents the set of all DDIs that can mediate a given set of PPIs for a given domain annotation scheme. As the underlying set of PPIs, we used a high-confidence yeast PPI data set created by the Interaction Detection Based On Shuffling (IDBOS) procedure at a 5% false discovery rate [20, 21].

Final DDI set	SET-1		SET-2		SET-3		SET-4		SET-5		SET-6	
	Num. of DDIs	Num. of PPIs	Num. of DDIs	Num. of PPIs	Num. of DDIs	Num. of PPIs	Num. of DDIs	Num. of PPIs	Num. of DDIs	Num. of PPIs	Num. of DDIs	Num. of PPIs
**PADDS**												
α = 0.0	3,995	51,270	3,826	92,196	3,057	300,254	3,071	296,195	3,025	335,638	2,619	420,499
α = 0.1	4,312	46,597	4,495	76,733	3,824	225,806	3,862	227,534	3,826	242,633	3,700	306,350
α = 0.2	4,372	46,556	4,598	75,956	3,877	221,263	3,927	224,804	4,018	225,233	3,790	304,560
α = 0.3	4,410	46,559	4,661	75,295	3,963	227,538	3,984	227,339	4,042	229,429	3,880	326,931
α = 0.4	4,466	46,544	4,699	76,241	3,988	224,914	3,966	224,986	4,183	221,487	3,940	325,481
α = 0.5	4,477	46,464	4,704	75,934	3,987	222,942	4,032	230,414	4,199	218,723	3,936	311,479
α = 0.6	4,494	46,486	4,701	77,593	4,030	226,433	4,025	224,024	4,350	225,464	3,958	311,333
α = 0.7	4,507	46,532	4,707	77,672	4,049	221,979	4,063	228,308	4,253	221,202	3,975	312,433
α = 0.8	4,508	46,560	4,716	77,615	4,097	224,943	4,128	214,496	4,287	223,465	3,984	312,390
α = 0.9	4,509	46,534	4,713	78,152	4,104	224,319	4,174	227,505	4,300	220,913	4,107	306,784
α = 1.0	4,514	46,486	4,704	77,986	4,050	224,393	4,153	225,682	4,344	216,809	4,084	304,639
CORE	3,807	43,120	3,319	62,038	2,326	161,394	2,390	154,364	1,932	163,463	1,814	254,342
**MSSC**												
MSSC	4,662	46,524	5,005	73,094	5,189	197,838	5,222	202,725	6,000	164,110	5,988	287,918
**GPE**												
GPE	8,930	67,198	7,971	130,543	5,681	369,018	5,555	370,689	8,057	408,453	5,908	489,181
**ALL POSSIBLE DDIs**												
ALL	8,930	67,198	12,887	147,724	14,078	402,596	13,791	404,366	36,834	466,853	30,527	528,045

Table S4 – Additional statistics of the extracted sets of domain-domain interactions using different methods and different protein-domain annotation sets.

Sets are defined as in Table 2 of the main text. Methods used to extract DDIs: the *parameter-dependent DDI selection* (PADDS), *maximum-specificity set cover* method (MSSC) [7], and the *generalized parsimonious explanation* (GPE) [8]. "ALL POSSIBLE DDIs" represents the set of all DDIs that can mediate a given set of PPIs for a given domain annotation scheme. As the underlying set of PPIs, we used a high-confidence yeast PPI data set created by the Interaction Detection Based On Shuffling (IDBOS) procedure at a 5% false discovery rate [20, 21].

Final DDI set	SET-1		SET-2		SET-3		SET-4		SET-5		SET-6	
	Precision	F-score	Precision	F-score	Precision	F-score	Precision	F-score	Precision	F-score	Precision	F-score
**PADDS**												
α = 0.0	0.14	0.24	0.08	0.16	0.03	0.05	0.03	0.05	0.02	0.05	0.02	0.04
α = 0.1	0.15	0.26	0.10	0.18	0.03	0.07	0.04	0.07	0.03	0.07	0.03	0.05
α = 0.2	0.15	0.26	0.10	0.19	0.04	0.07	0.04	0.07	0.04	0.07	0.03	0.05
α = 0.3	0.15	0.26	0.10	0.19	0.04	0.07	0.04	0.07	0.04	0.07	0.03	0.05
α = 0.4	0.15	0.26	0.10	0.19	0.04	0.07	0.04	0.07	0.04	0.07	0.03	0.05
α = 0.5	0.15	0.26	0.10	0.19	0.04	0.07	0.04	0.07	0.04	0.07	0.03	0.05
α = 0.6	0.15	0.26	0.10	0.18	0.04	0.07	0.04	0.07	0.04	0.07	0.03	0.05
α = 0.7	0.15	0.26	0.10	0.18	0.04	0.07	0.04	0.07	0.04	0.07	0.03	0.05
α = 0.8	0.15	0.26	0.10	0.18	0.04	0.07	0.04	0.07	0.04	0.07	0.03	0.05
α = 0.9	0.15	0.26	0.10	0.18	0.04	0.07	0.04	0.07	0.04	0.07	0.03	0.05
α = 1.0	0.15	0.26	0.10	0.18	0.04	0.07	0.04	0.07	0.04	0.07	0.03	0.05
CORE	0.15	0.26	0.10	0.18	0.04	0.07	0.04	0.07	0.04	0.06	0.02	0.05
**MSSC**												
MSSC	0.15	0.26	0.12	0.19	0.04	0.08	0.04	0.08	0.05	0.09	0.03	0.06
**GPE**												
GPE	0.10	0.19	0.06	0.11	0.02	0.04	0.02	0.04	0.02	0.04	0.02	0.03
**ALL POSSIBLE DDIs**												
ALL	0.10	0.19	0.05	0.10	0.02	0.04	0.02	0.04	0.02	0.03	0.02	0.03

References

1. Raghavachari B, Tasneem A, Przytycka TM, Jothi R: **DOMINE: a database of protein domain interactions**. *Nucleic Acids Res* 2008, **36**(Database issue):D656-661.
2. Yellaboina S, Tasneem A, Zaykin DV, Raghavachari B, Jothi R: **DOMINE: a comprehensive collection of known and predicted domain-domain interactions**. *Nucleic Acids Res* 2011, **39**(Database issue):D730-735.
3. Finn RD, Marshall M, Bateman A: **iPfam: visualization of protein-protein interactions in PDB at domain and amino acid resolutions**. *Bioinformatics* 2005, **21**(3):410-412.
4. Stein A, Panjkovich A, Aloy P: **3did Update: domain-domain and peptide-mediated interactions of known 3D structure**. *Nucleic Acids Res* 2009, **37**(Database issue):D300-304.
5. Stein A, Russell RB, Aloy P: **3did: interacting protein domains of known three-dimensional structure**. *Nucleic Acids Res* 2005, **33**(Database issue):D413-417.
6. Riley R, Lee C, Sabatti C, Eisenberg D: **Inferring protein domain interactions from databases of interacting proteins**. *Genome Biol* 2005, **6**(10):R89.
7. Huang C, Morcos F, Kanaan SP, Wuchty S, Chen DZ, Izaguirre JA: **Predicting protein-protein interactions from protein domains using a set cover approach**. *IEEE/ACM Trans Comput Biol Bioinform* 2007, **4**(1):78-87.
8. Guimaraes KS, Przytycka TM: **Interrogating domain-domain interactions with parsimony based approaches**. *BMC Bioinformatics* 2008, **9**:171.
9. Bru C, Courcelle E, Carrere S, Beausse Y, Dalmar S, Kahn D: **The ProDom database of protein domain families: more emphasis on 3D**. *Nucleic Acids Res* 2005, **33**(Database issue):D212-215.
10. Finn RD, Mistry J, Tate J, Coggill P, Heger A, Pollington JE, Gavin OL, Gunasekaran P, Ceric G, Forslund K *et al*: **The Pfam protein families database**. *Nucleic Acids Res* 2010, **38**(Database issue):D211-222.
11. Gough J, Karplus K, Hughey R, Chothia C: **Assignment of homology to genome sequences using a library of hidden Markov models that represent all proteins of known structure**. *J Mol Biol* 2001, **313**(4):903-919.
12. Haft DH, Loftus BJ, Richardson DL, Yang F, Eisen JA, Paulsen IT, White O: **TIGRFAMs: a protein family resource for the functional identification of proteins**. *Nucleic Acids Res* 2001, **29**(1):41-43.
13. Letunic I, Doerks T, Bork P: **SMART 6: recent updates and new developments**. *Nucleic Acids Res* 2009, **37**(Database issue):D229-232.
14. Marchler-Bauer A, Lu S, Anderson JB, Chitsaz F, Derbyshire MK, DeWeese-Scott C, Fong JH, Geer LY, Geer RC, Gonzales NR *et al*: **CDD: a Conserved Domain Database for the functional annotation of proteins**. *Nucleic Acids Res* 2011, **39**(Database issue):D225-229.
15. Schultz J, Milpetz F, Bork P, Ponting CP: **SMART, a simple modular architecture research tool: identification of signaling domains**. *Proc Natl Acad Sci U S A* 1998, **95**(11):5857-5864.
16. Hart GT, Ramani AK, Marcotte EM: **How complete are current yeast and human protein-interaction networks?** *Genome Biol* 2006, **7**(11):120.

17. Sambourg L, Thierry-Mieg N: **New insights into protein-protein interaction data lead to increased estimates of the S. cerevisiae interactome size**. *BMC Bioinformatics* 2010, **11**:605.
18. von Mering C, Krause R, Snel B, Cornell M, Oliver SG, Fields S, Bork P: **Comparative assessment of large-scale data sets of protein-protein interactions**. *Nature* 2002, **417**(6887):399-403.
19. Yu H, Braun P, Yildirim MA, Lemmens I, Venkatesan K, Sahalie J, Hirozane-Kishikawa T, Gebreab F, Li N, Simonis N *et al*: **High-quality binary protein interaction map of the yeast interactome network**. *Science* 2008, **322**(5898):104-110.
20. Yu X, Ivanic J, Memisevic V, Wallqvist A, Reifman J: **Categorizing biases in high-confidence high-throughput protein-protein interaction data sets**. *Mol Cell Proteomics* 2011, **10**(12):M111 012500.
21. Yu X, Ivanic J, Wallqvist A, Reifman J: **A novel scoring approach for protein co-purification data reveals high interaction specificity**. *PLOS Computational Biology* 2009, **5**(9):e1000515.
